# Coupling Taguchi experimental designs with deep adaptive learning enhanced AI process models for experimental cost savings in manufacturing process development

**DOI:** 10.1038/s41598-024-73669-1

**Published:** 2024-10-08

**Authors:** Syed Wasim Hassan Zubair, Syed Muhammad Arafat, Sarmad Ali Khan, Sajawal Gul Niazi, Muhammad Rehan, Muhammad Usama Arshad, Nasir Hayat, Tauseef Aized, Ghulam Moeen Uddin, Fahid Riaz

**Affiliations:** 1 Department of Mechanical Engineering, University of Engineering & Technology, Lahore, 54890 Pakistan; 2 Department of Mechanical Engineering, Faculty of Engineering & Technology, The University of Lahore, Lahore, 54000 Pakistan; 3 Department of Industrial and Manufacturing Engineering, University of Engineering and Technology, Lahore, 54890 Pakistan; 4 School of Mechanical and Electrical Engineering, University of Electronic Science and Technology of China, Chengdu, 611731 Sichuan China; 5 Center for System Reliability and Safety, University of Electronic Science and Technology of China, Chengdu, 611731 Sichuan China; 6 College of Engineering, Abu Dhabi University, Abu Dhabi, UAE

**Keywords:** Artificial Intelligence, Taguchi, Dry Finishing, Turning, Aerospace Alloy, Neural networks, Support Vector machines, Decision trees, Aerospace engineering, Mechanical engineering, Environmental impact

## Abstract

The Aluminum alloy AA7075 workpiece material is observed under dry finishing turning operation. This work is an investigation reporting promising potential of deep adaptive learning enhanced artificial intelligence process models for L_18_ (6^1^3^3^) Taguchi orthogonal array experiments and major cost saving potential in machining process optimization. Six different tool inserts are used as categorical parameter along with three continuous operational parameters i.e., depth of cut, feed rate and cutting speed to study the effect of these parameters on workpiece surface roughness and tool life. The data obtained from special L_18_ (6^1^3^3^) orthogonal array experimental design in dry finishing turning process is used to train AI models. Multi-layer perceptron based artificial neural networks (MLP-ANNs), support vector machines (SVMs) and decision trees are compared for better understanding ability of low resolution experimental design. The AI models can be used with low resolution experimental design to obtain causal relationships between input and output variables. The best performing operational input ranges are identified for output parameters. AI-response surfaces indicate different tool life behavior for alloy based coated tool inserts and non-alloy based coated tool inserts. The AI-Taguchi hybrid modelling and optimization technique helped in achieving 26% of experimental savings (obtaining causal relation with 26% less number of experiments) compared to conventional Taguchi design combined with two screened factors three levels full factorial experimentation.

## Introduction

The aluminum alloys are widely used for a variety of applications and amongst the second largest in use alloys after steel due to high strength to weight ratio^1^. The selection of alloying elements depends upon the application as each alloying element imparts different characteristics in the material^2^. Aluminum alloy AA7075 has gained a prominent prospect in industry specially in last few decades owed to its remarkable properties e.g., high strength to weight ratio, high mechanical strength and low corrosion rate etc^3,4^. . These characteristics makes AA7075 suitable for variety of industries like automotive industry, manufacturing industry, naval or sea water application industry and aerospace industry etc. Cerchier et al., worked with AA7075 for sea water and naval applications after copper coating through plasma electrolytic oxidation studying anti-biofouling and anticorrosive properties^5^. Andreatta et al., investigated promising corrosion properties of AA7075 by varying heat treatment parameters^6^. Karabay et al., investigated the erosion behavior of AA7075 for industrial application^7^. Ramkumar et al., worked with the metal matrix composite of AA7075 and TiC with ceramic particles to enhance hardness, elastic modulus and creep resistance to a ductile metallic phase that restrict major brittleness flaw in ceramics^8^. An important investigation for automotive and aerospace industry comprise of machining AA7075. The selection of right machining performance parameters and related operational parameters are directly related to cost of machining and in turn cost of product. The aluminum alloys are considered as difficult to machine materials due to the machining chips adhesion on tool insert, build-up edges (BUE) and material diffusion etc^9,10^. In addition to optimizing the machining parameters the machining cost can also be reduced by dry machining process^11,12^.The minimum quantity liquid (MQL) utilizes low fluid amount. However, it still requires particular equipment and fluid management. Extra cost is associated with both flooded and MQL during installation (tanks, pumps, hoses and disposal systems etc.), service (liquid cost, operational/maintenance cost and handling cost) and lubricant disposal^13^. The environmental dangers associated with the use of lubricants (e.g., lubricant disposal and fume exposure to operators) and added complexity to machining process (e.g., handling and applying lubricant) can be completely avoided by dry machining process^11,12,14,15^. The dry machining is a simple and clean process. Factors like adherence to stringent environmental and occupational health and safety standards, manufacturing cost saving and advancements in tool inserts materials and technology favor the use of dry machining in industries like automotive industry, manufacturing industry, naval or sea water application industry and aerospace industry etc.

Dry machining requires few fundamental changes in the machining setup and one of the most important change is selection of right tool insert^16–18^. The ceramic coatings especially titanium based ceramic coatings offers self-solid lubrication properties due to the formation of TiO_2_ that can help in dry machining process^19^. Kara et al., used ceramic coated tools in dry machining and investigated the effect of deep cryogenic treatment of AISI D2 steel on dry machining performance parameters i.e., tool life and surface roughness^20^. Marousi et al., worked with titanium metal matrix composites in dry machining operation using physical vapor deposition based ceramic (TiN/TiAlN multilayers coating) coated carbide tool^21^.

The industries with high-tech applications (e.g., aerospace and automotive industry) require state of art engineered components with minimum cost possible. The selection of tool life as performance parameter is directly related to cost of the product made. Marousi et al., investigated tool life of ceramic coated carbide tool insert in dry machining operation^21^. Cavaleiro et al., argued that to increase the tool life in dry machining process tools inserts needs to be coated with coatings having self-lubricating ability^22^. Iqbal et al., stress on increasing the tool life and hence reducing the production cost for sustainable and responsible machining practices^23^. Resulting workpiece surface finish is another important factor and can be considered as a performance parameter in dry machining process due to its direct relation with product quality. Das et al., performed full factorial experiments with TiN coated Al_2_O_3_ + TiCN mixed ceramic inserts in dry turning operation and concluded that the resulting workpiece surface roughness and tool life is related to product quality and production cost^24^. Padhan et al., worked on machining sustainability assessment using cooling/lubrication techniques e.g., flooded, minimum quality lubricant, compressed air cooled and dry. The machining work was done by taking tool life and surface finish among measured experimental responses^25^.

The selection of operational parameters with rightly configured combination is an important criterion especially for dry finishing machining operations^26–28^. According to previous research work, for dry finishing turning operations depth of cut (DOC), feed rate (FR) and cutting speed (CS) are critical operational parameters^24–28^.

The cost both in terms of money and time associated with the number of experiments; required to fully understand the causal behavior of input and output variables, can be staggering some times. Moreover, experiments need to be designed first, rather than randomly performing or having to change one factor at a time (OFAT)^29^. Taguchi design of experiments performs exceptionally well for finding out the main effects in an experimental study. However, the information between interaction may be lost or aliased in main effects depending upon the Taguchi experimental design. Therefore, caution should be practiced in creating, analyzing and drawing conclusion from Taguchi design of experiments^30^.

Machine learning methods are significantly gaining attention in every area of science and technology and are very effective in drawing causal relationships between input and output parameters. Different machine learning methods are applied successfully on large data volumes. However, studies have also showed that the machine learning methods can also learn complex relationships between data with lesser or few data points^20,31,32^. Although, machine learning showed its prominence in every field of science, its use in machining is very restricted. Kara et al., used artificial neural network to evaluate surface roughness and trained it with 162 data points. Different learning algorithms were trained to find out best possible match for surface roughness^20^. Limited amount of research work available investigating machining parameters using machine learning tools. The discussion on Taguchi-machine learning approach needs a comprehensive investigation especially for machining applications.

This research work aims to investigate whether the compromise made by reducing number of experiments in Taguchi experimental design can be accommodated by machine learning tools. The effect of hyper-parameter optimization and dimensional changes on low resolution experimental design is analyzed. Artificial Neural Networks (ANNs), Support Vector Machines (SVMs) and Decision Trees are the selected deep learning AI tools. The training data is provided through special L_18_ (6^1^3^3^) orthogonal array experimental design. Tool life and resulting workpiece surface roughness are taken as dry finishing turning performance parameters and tool insert type, depth of cut (DOC), feed rate (FR) and cutting speed (CS) are taken as corresponding operational parameters. The results are discussed on the basis of goodness of fit criteria and external validation.

## Methodology

### Experimental details

This research work focuses on the development of AI-Taguchi hybrid approach for machining process development to model and mine nonlinearity and aliasing information from low resolution Taguchi orthogonal array experimental design. The approach can open new possibilities for industry to achieve low cost data driven manufacturing process development solutions. The finishing turning operation machining data set used for training AI models is taken from recently published research work^33^ and proposes a low experimental cost AI-Taguchi hybridized modeling approach. Zubair et al., used conventional regression techniques for data analysis^33^. The experimental details are briefly explained for the development of better understanding in current section.

#### Dry finishing turning operation

**Fig. 1:**
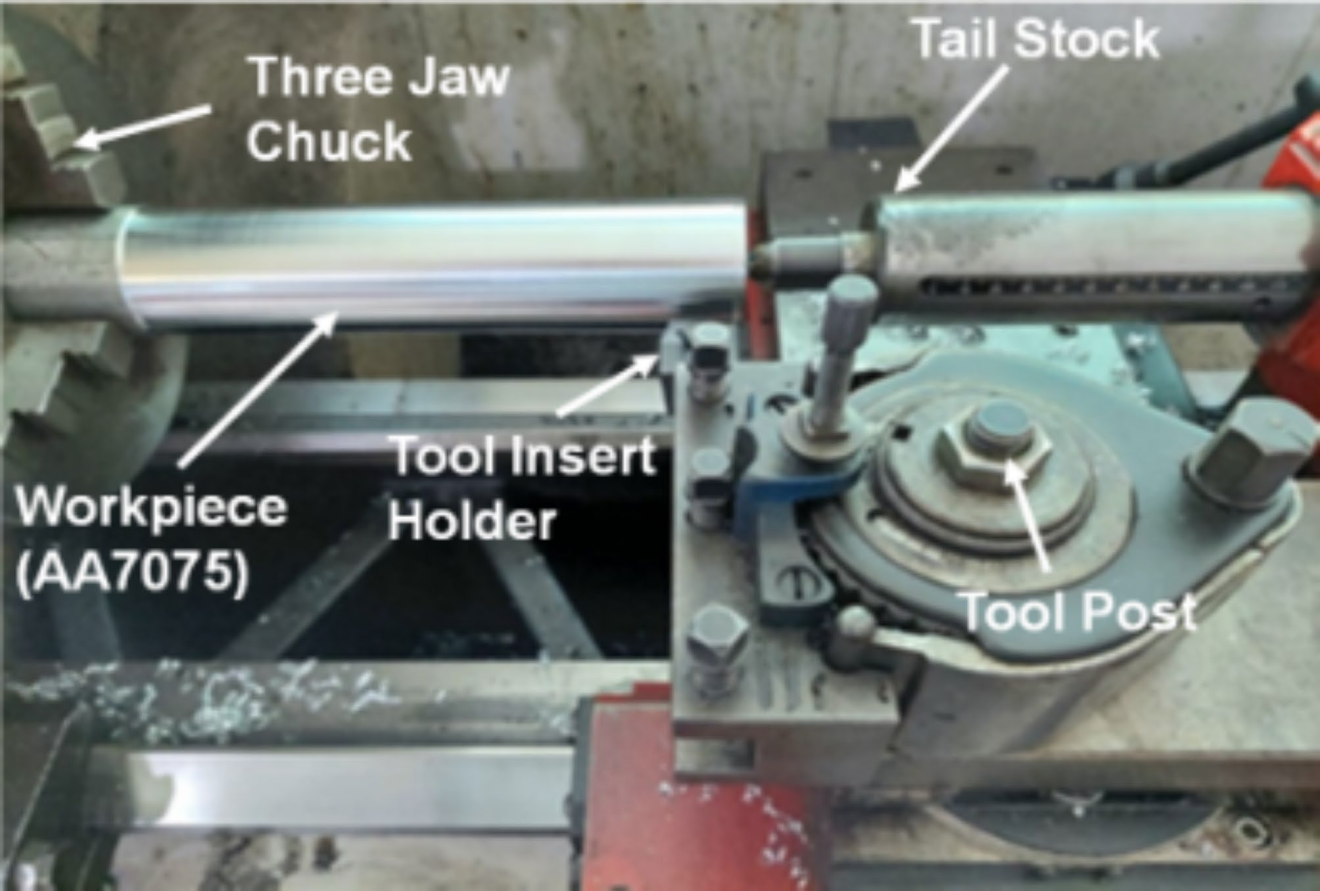
Workpiece configuration on precision lathe machine.

The dry finishing turning operation is performed on aerospace grade aluminum alloy (AA 7075). The workpiece material (AA 7075) is dimensioned as 2438.4-mm long length of 50.8-mm diameter circular bars. All workpieces are kept at constant dimension at the start of each experimental setting to keep experimental uniformity. The machining is performed on precision lathe machine EMCOMAT 17D having a Digital Read Out (DRO) for numeric display of spindle RPM and tool positioning in three dimensions (X, Y and Z axis). It is critical to perform vibration free turning operation to abstain from vibration based erroneous readings therefore, head stock and tail stock are engaged in turning operation. The workpiece configuration during experimentation is shown in Fig. 1.

The study is conducted to evaluate the effect of tool inserts selection and operational parameters on the performance parameters in dry finishing turning operation of AA 7075. One uncoated and five coated tungsten carbide (WC) tool inserts are analyzed for their effect on studied output parameters. The tool inserts coating details are given in Table 1.

**Table 1:** Details of tool inserts used in experimentation.

No.	ISO number	Designated name	Type of coating	Source
1	SNMG 120,408 (Tungsten Carbide (WC) Tool Insert)	Insert 1	Uncoated	PVD coated from market
2		Insert 2	Titanium Nitride (TiN)	
3		Insert 3	Titanium Carbo Nitride (TiCN)	
4		Insert 4	Titanium Aluminum Nitride (TiAlN (50:50))	Custom coated through PVD process using novel alloy targets
5		Insert 5	Titanium Aluminum Nitride (TiAlN (70:30))	
6		Insert 6	Titanium Aluminum Nitride (TiAlN (80:20))	

**Fig. 2:**
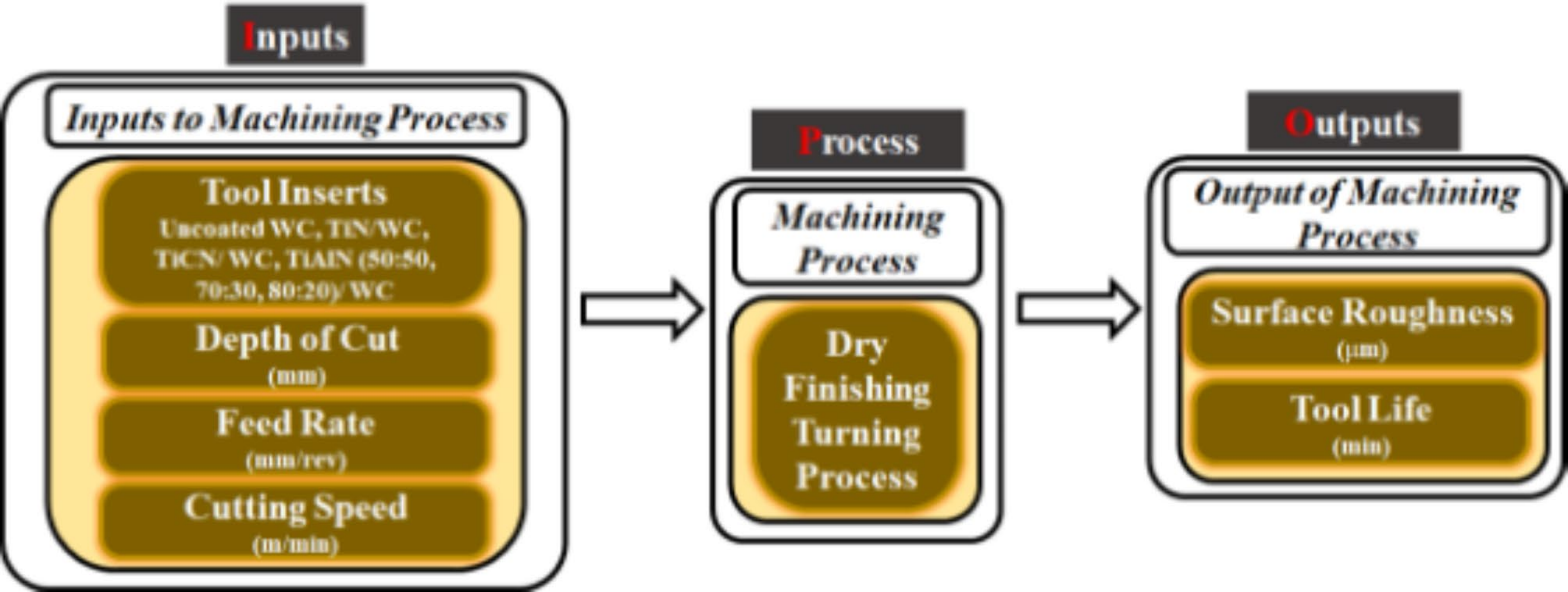
IPO diagram for dry finishing turning process.

The operational parameters consist of depth of cut (DOC), feed rate (FR) and cutting speed (CS). These operational parameters are selected on the basis of available literature^34–36^. The levels of operational parameters are selected considering the requirement of finishing operation i.e., the surface roughness needs to be minimized. Turning operation is carried out in dry conditions (without coolant) to minimize environmental hazards and operational cost. Tool life and surface roughness are the selected performance parameters for dry finishing turning operation. The input-process-output (IPO) diagram for dry finishing turning process is given in Fig. 2.

#### Physical vapor deposition (PVD)

Three custom ceramic coatings are developed for WC tool inserts through cathodic arc physical vapor deposition method. Alloy targets with different composition i.e., Ti_50_Al_50_, Ti_70_Al_30_ and Ti_80_Al_20_ are used to obtain novel TiAlN(50:50), TiAlN(70:30) and TiAlN(80:20) coatings on WC tool inserts respectively. Bare WC is initially cleaned ultrasonically using TCE (trichloroethylene). The cleaning process is followed by drying process in an electric oven. The uncoated WC tool inserts are then carefully mounted into PVD chamber. A vacuum of 1 × 10^− 5^ mbar is created through vacuum pumps in PVD chamber^37,38^. All WC tool inserts are coated for 90 min at 300 °C. Bias voltage and target current is set at 250 V and 10 Amps respectively. The coating process and post-coating cooling process is carried out in argon (Ar) and nitrogen (N_2_) environment.

#### Measurement of tool life and surface roughness

Machining time (T_mn_) for nth cut is measured by taking turning length (L_n_) for nth cut, feed rate and spindle speed (N) as given in Eq. (1)^33,39,40^. Total tool life (T_L_) for an experiment is calculated by summation of machining times for 1 to nth cuts obtained until the stopping criteria is reached i.e., reaching flank wear value of 150 μm. Equation (2) represents total tool life (T_L_) measured for an experiment.

1 $$\:{T}_{mn}=\frac{{L}_{n}}{f.N}$$

2 $$\:{T}_{L}=\sum\:_{i=1}^{n}{T}_{mi}$$

The surface roughness is calculated by using Insize ISR-1000B roughness tester with 0.001 μm resolution and ± 3% accuracy. A total of five measurements are taken on different points of workpiece at the end of each experiment. Each reading is taken with 1 mm/s speed and 0.8 mm length. Average value is then taken as resulting workpiece surface roughness. Equation (3) indicates reported arithmetic mean surface roughness (Ra)^16,41,42^.

3 $$\:{R}_{a}=\frac{1}{L}{\int\:}_{0}^{L}\left|Z\right|dx$$

### Experimental design and AI integration

The experiments are conducted with low resolution Taguchi L_18_ (6^1^3^3^) orthogonal array experimental design. Artificial intelligence models are used to evaluate their efficacy with highly reduced experimental dimensions. The effect of hyper-parameter optimization or change in AI model dimensions is studied for compensation of reduced experimental dimensions.

#### Experimental design

The selection of experimental design is one of the most critical element in an experimental study that directly relates with quality of information gathered, cost and time consumed on experiments. It is important to select the experimental design keeping in view the number and levels of independent variables^43,44^. This research work includes both categorical (Tool inserts) and continuous variables (depth of cut (DOC), feed rate (FR) and cutting speed (CS)). The information about main effects and all possible interaction combination can be obtained by full factorial experimental design. Tool insert is a six level factor and DOC, FR and CS have three levels each. A full factorial experimental design would contain 6 × 3 × 3 × 3 = 162 experiments. This high resolution experimental design leads to a very high resource utilization. The experimental cost is reduced by creating a low resolution experimental design. However, the information obtained in this type of design is aliased due to limitation in degree of freedom. A special L_18_ (6^1^3^3^) orthogonal array experimental (Taguchi) design is selected for analysis, reducing the number of experiments from 162 (for full factorial design) to 18. The selection of further reduced resolution experimental configuration could lead to an unbalanced design. Moreover, if possible it is advisable to plan reduced resolution experimental design in such a way that main effects along with some interaction information can be obtained using conventional analysis technique (considering experimental design degree of freedom)^30,45,46^. This can be obtained using selected L_18_ (6^1^3^3^) orthogonal array experimental design for this particular study. The selection of increased resolution experimental design compared to L_18_ (6^1^3^3^) leads to additional cost and effort. Table 2 represents level details, experimental combination and run order for special L_18_ (6^1^3^3^) orthogonal array experimental design. The repeatability analysis is performed on 33% of selected experiments to ensure the validity of experimental setup.

**Table 2:** Special L_18_ (6^1^3^3^) orthogonal array experimental design.

Experiment No.	Run order	Tool insert type	DOC (mm)	FR (mm/rev)	CS (m/min)
1	11	Uncoated WC (Level 1)	0.1 (Level 1)	0.112 (Level 1)	100 (Level 1)
2	2	Uncoated WC (Level 1)	0.2 (Level 2)	0.225 (Level 2)	150 (Level 2)
3	15	Uncoated WC (Level 1)	0.3 (Level 3)	0.337 (Level 3)	200 (Level 3)
4	13	TiN coated on WC (Level 2)	0.1 (Level 1)	0.112 (Level 1)	150 (Level 2)
5	17	TiN coated on WC (Level 2)	0.2 (Level 2)	0.225 (Level 2)	200 (Level 3)
6	12	TiN coated on WC (Level 2)	0.3 (Level 3)	0.337 (Level 3)	100 (Level 1)
7	4	TiCN coated WC (Level 3)	0.1 (Level 1)	0.225 (Level 2)	100 (Level 1)
8	1	TiCN coated WC (Level 3)	0.2 (Level 2)	0.337 (Level 3)	150 (Level 2)
9	10	TiCN coated WC (Level 3)	0.3 (Level 3)	0.112 (Level 1)	200 (Level 3)
10	7	TiAlN(50:50) coated WC (Level 4)	0.1 (Level 1)	0.337 (Level 3)	200 (Level 3)
11	8	TiAlN(50:50) coated WC (Level 4)	0.2 (Level 2)	0.112 (Level 1)	100 (Level 1)
12	3	TiAlN(50:50) coated WC (Level 4)	0.3 (Level 3)	0.225 (Level 2)	150 (Level 2)
13	9	TiAlN(70:30) coated WC (Level 5)	0.1 (Level 1)	0.225 (Level 2)	200 (Level 3)
14	5	TiAlN(70:30) coated WC (Level 5)	0.2 (Level 2)	0.337 (Level 3)	100 (Level 1)
15	16	TiAlN(70:30) coated WC (Level 5)	0.3 (Level 3)	0.112 (Level 1)	150 (Level 2)
16	6	TiAlN(80:20) coated WC (Level 6)	0.1 (Level 1)	0.337 (Level 3)	150 (Level 2)
17	14	TiAlN(80:20) coated WC (Level 6)	0.2 (Level 2)	0.112 (Level 1)	200 (Level 3)
18	18	TiAlN(80:20) coated WC (Level 6)	0.3 (Level 3)	0.225 (Level 2)	100 (Level 1)

#### Artificial neural network (ANN)

The multilayer perceptron based artificial neural network (MLP-ANN) has ability to approximate functions or complex relations between input and output. The MLP-ANN models are independent of degree of freedom. Therefore, these models are investigated for the ability to mine/map compromised or lost information in low resolution experimentation. The approach to use MLP-ANN models (or in broader spectrum machine learning models) to compensate experimental compromise is almost non-existent in literature. MLP-ANN are known for their ability to map complex non-linarites present in a system^47,48^. The data obtained from performing special L_18_ (6^1^3^3^) orthogonal array experiments are used to train fully connected MLP-ANN models. It is expected that the trained models can predict significant main effects and interactions between operational parameters (tool inserts, DOC, FR, CS) and their corresponding effect on performance parameters (i.e., tool life & surface roughness). Separate models are trained for tool life and surface roughness using scaled conjugate gradient based back propagation algorithm. Decision about number of neurons in a hidden layer or number of hidden layers in a model is always a tricky one. Usually, this decision is taken by trial and error method^49,50^. This problem is solved by a comprehensive model design with 1–4 hidden layers and 4,6,8,10 and 12 neurons. The models are designed to spread both horizontally and vertically. A total of 780 models are trained for tool life and similarly, 780 separate models for surface roughness. The comprehensive model design serves two fold (a) not using trial and error method for selecting a network configuration and relying on data (b) the effect study of horizontal and vertical dimensional spread in MLP-ANN on result interpretation of low dimension experimental design i.e., special L_18_ (6^1^3^3^) orthogonal array experiments. The tangent hyperbolic transfer function is used for both input- hidden layer and hidden-output layer. Data split of training, testing and validation is 70%, 15% and 15% respectively. The MLP-ANN model design is shown in Tables 3 and 4.

**Table 3:** MLP-ANN total trained models.

Slab no	Number of layers involved	Levels of neurons	Number of models	Totals models
1	1	4,6,8,10,12 (5 Levels)	5	780
2	2		25	
3	3		125	
4	4		625	

**Table 4:** MLP-ANN layer and neuron configuration for each model.

Model no.	Layer 1 Neurons	Layer 2 Neurons	Layer 3 Neurons	Layer 4 Neurons
1	4	**–**	**–**	**–**
2	6	**–**	**–**	**–**
3	8	**–**	**–**	**–**
.	.	.	.	.
.	.	.	.	.
.	.	.	.	.
28	12	8	**–**	**–**
29	12	10	**–**	**–**
30	12	12	**–**	**–**
.	.	.	.	.
.	.	.	.	.
.	.	.	.	.
106	10	4	4	**–**
107	10	4	6	**–**
108	10	4	8	**–**
.	.	.	.	.
.	.	.	.	.
.	.	.	.	.
778	12	12	12	8
779	12	12	12	10
780	12	12	12	12

#### Support vector machine (SVM)

The support vector machine is selected due to its remarkable ability to perform on classification problems. The current problem discussed in this research work has characteristics of both classifier (due to tool insert as categorical variable) and continuous function (due to DOC, FR, CS, tool life and surface roughness as continuous variables). Moreover, the SVM model is developed structurally to convert a low dimension input-output space into high dimension input-output space by transformation. This can be beneficial, specially with low resolution/dimensional experimentation. Equation (4) gives a simplified representation of space transformation^51,52^.

4 $$\:K\left({x}_{i},{x}_{j}\right)=\:\varPhi\:{\stackrel{-}{x}}_{\dot{i}}.\varPhi\:{\stackrel{-}{x}}_{\dot{j}}$$

Where, kernel function is *k* and mapping function is ϕ. Another advantage of SVM model is that it is also independent of degree of freedom however, usually large data is required to properly train for complex relations. The data obtained from performing special L_18_ (6^1^3^3^) orthogonal array experiments are used to train SVM models.

The optimizable and medium Gaussian SVM architectures are trained for both tool life and surface roughness performance parameters. Two optimizer i.e.; Bayesian optimization and random search, are used with linear, quadratic, cubic and Gaussian kernel functions. The hyper-parameters optimization is one of the critical process for the assurance of having a generalized solution. The hyper-parameters give penalty strength (*c*) to SVM models enabling the models to usually map a generalized solution by avoiding overfitting. The range of hyper-parameters is set as 0.001–1000 and 0.0005-50 for box constraints and epsilon respectively. Maximum number of epochs is set to thirty for the training of SVM models. The details of SVMs are summarized in Table 5.

**Table 5:** SVM models and hyper-parameter details.

No.	Model	Optimizer	Kernel function	Optimized box constraint for TL	Optimized epsilon for TL	Optimized box constraint for SR	Optimized epsilon for SR
1	Optimizable SVM	Bayesian optimization	Gaussian	955.6318	0.38529	34.9258	0.0052294
2	Optimizable SVM	Bayesian optimization	Linear	17.9898	0.33227	58.042	0.056561
3	Optimizable SVM	Bayesian optimization	Quadratic	0.21079	0.00399	0.09488	0.11178
4	Optimizable SVM	Bayesian optimization	Cubic	10.4894	0.000492	0.046117	0.0036721
5	Optimizable SVM	Random Search	Gaussian	1.1987	0.39892	3.4328	0.31108
6	Optimizable SVM	Random Search	Linear	4.8329	0.28263	0.12907	0.065943
7	Optimizable SVM	Random Search	Quadratic	25.6595	0.26028	0.010857	0.0009375
8	Optimizable SVM	Random Search	Cubic	4.7785	0.014007	78.3815	8.4117
9	Medium Gaussian SVM	–	Gaussian	0.43746	0.04374	0.74346	0.07434
10	Medium Gaussian SVM	–	Linear	0.43746	0.04374	0.74346	0.07434
11	Medium Gaussian SVM	–	Linear	0.5	0.04374	0.5	0.07434

#### Trees

The decision tree method is used due to its ability to obtain solutions in scenario based situations^53,54^. The decision to choose particular type of tool insert with specific operational settings is an important one. The data obtained from performing special L_18_ (6^1^3^3^) orthogonal array experiments are used to train decision trees. The optimizable ensemble and bagged tree algorithms are trained. In optimizable ensemble model and bagged trees minimum leaf size is taken as 4 with 4,8 & 12 number of learners. Bayesian optimization with expected improvement per second plus as acquisition function is used for three optimizable ensemble models and random search is used for remaining three optimizable ensemble models.

#### Models evaluation criteria

MLP-ANN, SVM and Trees are evaluated on seven performances criteria. The coefficient of determination R^2^ is given in Eq. (5)^55,56^. The model R-squared value R^2^ = 0 has a very poor prediction ability similarly, R^2^ = 1 is considered as an over fit machine learning model. The mathematical expressions for root mean square error (RMSE), normalized root mean square error (NRMSE), mean absolute error (MAE) and mean absolute percentage error (MAPE) are given in Eqs. (6), (7), (8) and (9) respectively^57–59^. A smaller value for each error measure indicated better model performance. Nash and Sutcliffe efficiency (NSE) and modified agreement index (*d*) are given in Eqs. (10) and (11) respectively^55^. These measures find out the perfect match or closeness between actual and model predicted value.

5 $$\:{R}^{2}=1-\frac{{\sum\:}_{i=1}^{n}{\left({y}_{i}-{\widehat{y}}_{i}\right)}^{2}}{{\sum\:}_{i=1}^{n}{\left({y}_{i}-{\stackrel{-}{y}}_{i}\right)}^{2}\:}$$

6 $$\:RMSE=\sqrt{\frac{1}{n}\sum\:_{i=1}^{n}{\left({y}_{i}-{\widehat{y}}_{i}\right)}^{2}}$$

7 $$\:NRMSE=\frac{RMSE}{{y}_{max}-{y}_{min}}\times\:100\%$$

8 $$\:MAE=\frac{1}{n}\sum\:_{i=1}^{n}\left|{y}_{i}-{\widehat{y}}_{i}\right|$$

9 $$\:MAPE=\frac{1}{n}\sum\:_{i=1}^{n}\left|\frac{{\widehat{y}}_{i}-{y}_{i}}{{y}_{i}}\right|\times\:100\%$$

10 $$\:NSE=1-\frac{\sum\:_{i=1}^{N}\left|{y}_{i}-{\widehat{y}}_{i}\right|}{\sum\:_{i=1}^{N}\left|{y}_{i}-{\stackrel{-}{y}}_{i}\right|}$$

11 $$\:d=1-\frac{\sum\:_{i=1}^{N}\left|{y}_{i}-{\widehat{y}}_{i}\right|}{\sum\:_{i=1}^{N}\left|{y}_{i}-{\stackrel{-}{y}}_{i}\right|+\left|{\widehat{y}}_{i}-{\stackrel{-}{y}}_{i}\right|}$$

#### External validation

The external validation is done within inner bound of training data range. Only best performing deep learning technique is used for external validation. Dry finishing turning operations are performed on selected input configurations of operational parameters. Experimental results are then compared with predicted AI results to assess model performance on untrained data. The details of experimental settings for external validation are given in Table 6.

**Table 6:** Experiments selected for external validation.

No	Tool insert type	DOC (mm)	FR (mm/rev)	CS (m/min)
1	TiCN (3)	0.1	0.112	200
2	TiN (2)	0.1	0.112	200

**Fig. 3:**
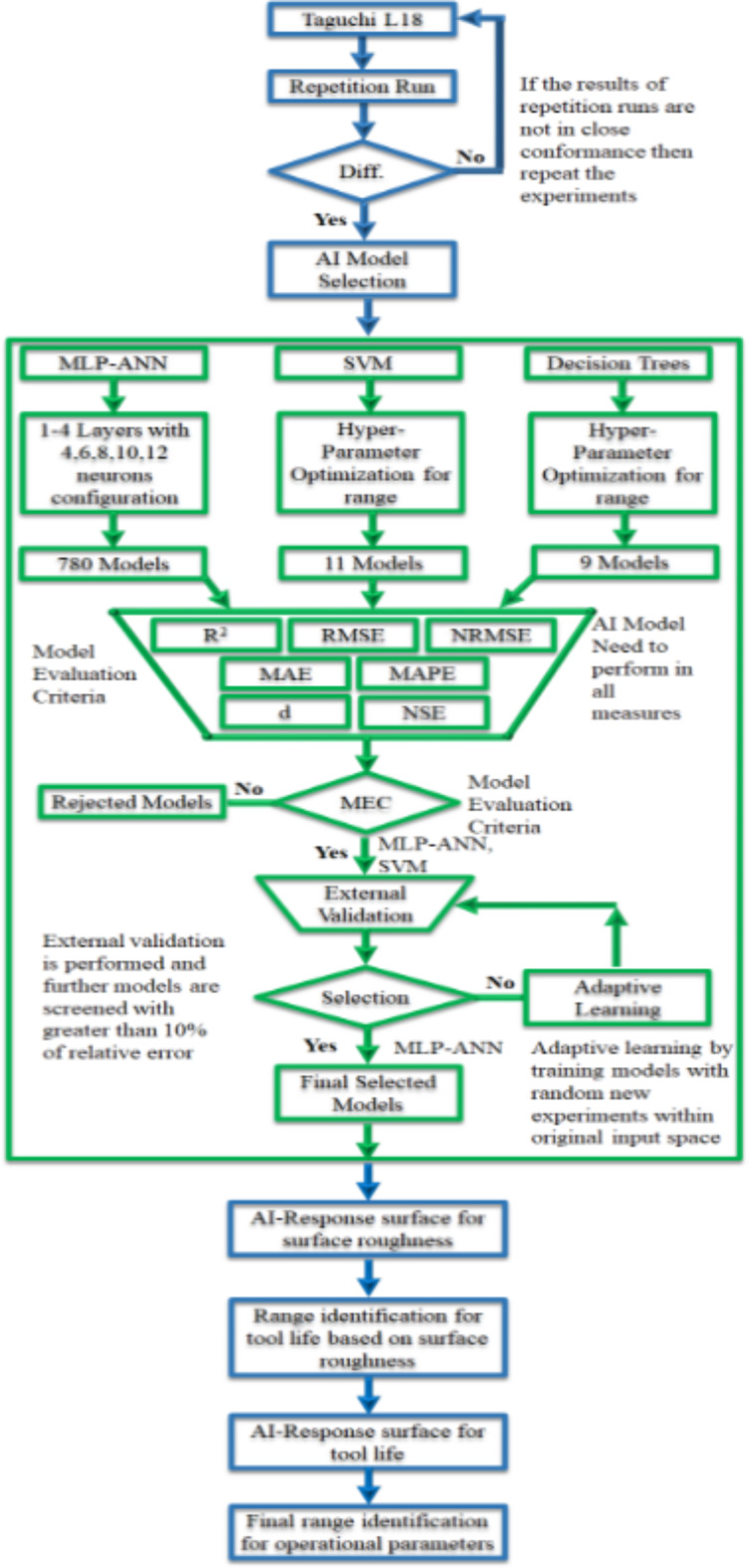
AI-Taguchi hybrid technique methodology diagram.

The AI-Taguchi hybrid technique used in this research work is summarized in methodology diagram given in Fig. 3.

#### Results and discussion

The results obtained in dry finishing turning operation of aluminum alloy AA7075 by implementing special L_18_ (6^1^3^3^) orthogonal array experimental design are analyzed in this section. Different machine learning algorithms are deployed to understand complex relations between inputs and outputs.

#### Tool life and surface roughness

**Fig. 4:**
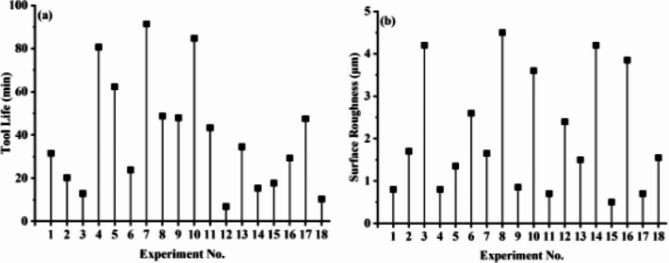
L_18_ (6^1^3^3^) orthogonal array experimental results (**a**) tool life (**b**) surface roughness.

**Fig. 5:**
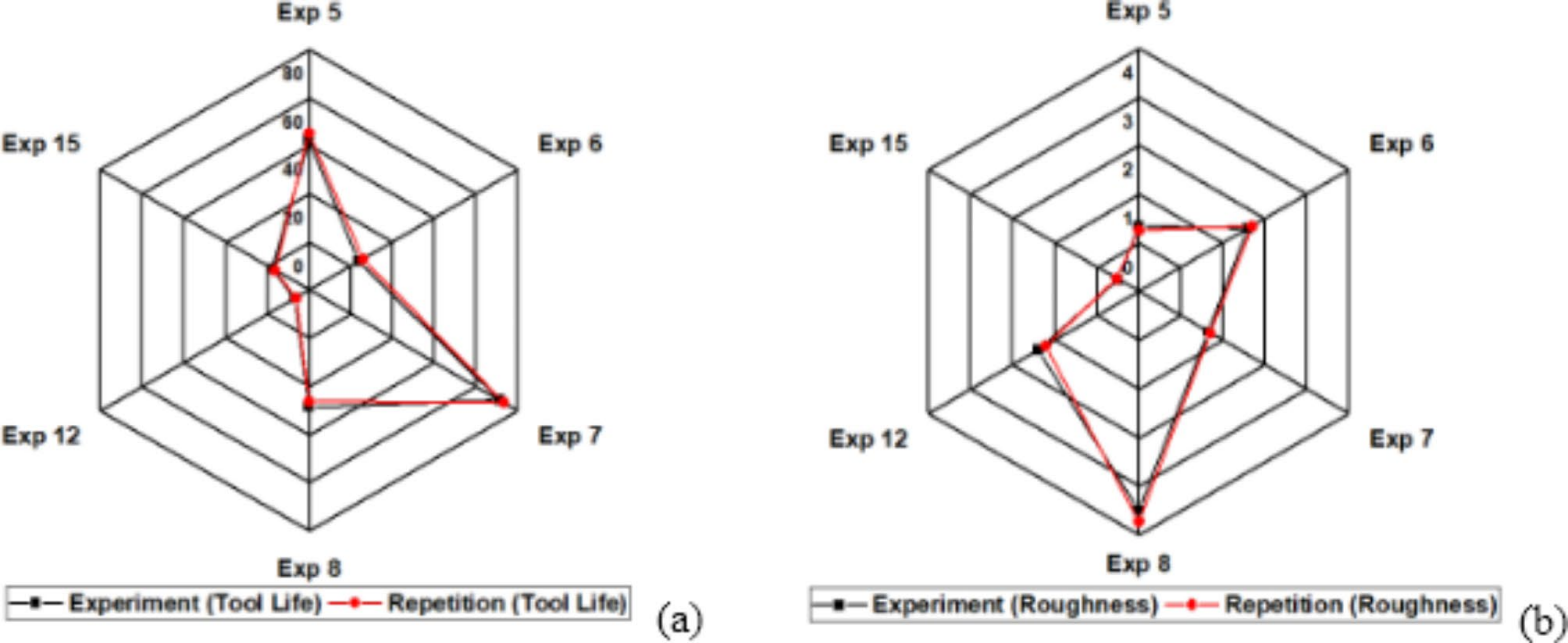
Experimental repeatability analysis (**a**) tool life (**b**) surface roughness.

The dry finishing turning experiments are performed on precision lathe machine. All experiments are performed in random order to reduce the effect of unknown factors or any human bias. Figure 4a, b represents tool life and resulting workpiece surface roughness of all special L_18_ (6^1^3^3^) orthogonal array experiments. To ensure the validity of experimental setup, experimental repeatability analysis is performed. The results of repeatability analysis are shown in Fig. 5. The results are in close conformance indicating validity of experiments. Figure 4a indicates that maximum tool life is obtained for TiCN coated WC tool insert in experiment 7 and minimum tool life is obtained for TiAlN(50:50) coated WC tool insert in experiment 12. Figure 4b indicates that maximum workpiece resulting surface finish is obtained for TiAlN(70:30) coated WC tool insert in experiment 15 and minimum observed for TiCN coated WC tool insert in experiment 8. A detailed observation of both Fig. 4a,b indicates that there exists no one to one relation between input and output parameters. A possibility of complex interactive behavior exists that can lead toward best possible tradeoff situation. This can be obtained by surface plots of interactive parameters with respect to performance parameters. Taguchi arrays are famous for digging out main effects and regression analysis is limited due to degree of freedom in this particular case. Therefore, it is not possible to obtain all interactions by using conventional analysis techniques.

#### Model evaluation and selection

The models are evaluated on the basis of criteria discussed in Section “Models evaluation criteria”. Best models are selected having greater than 0.85 R^2^ with the combination of other parameters i.e., RMSE < 0.17, NRMSE < 10, MAE < 0.1, MAPE < 4, NSE > 0.75, d > 0.85. The parameters with maximum range are not selected as this can lead to a model with overfitting. The generalized models are more suitable as these models can predict with reasonable efficiency in scenarios outside of training data set. The model generalization is also ensured through external validation of the selected models.

#### MLP-ANN

**Fig. 6:**
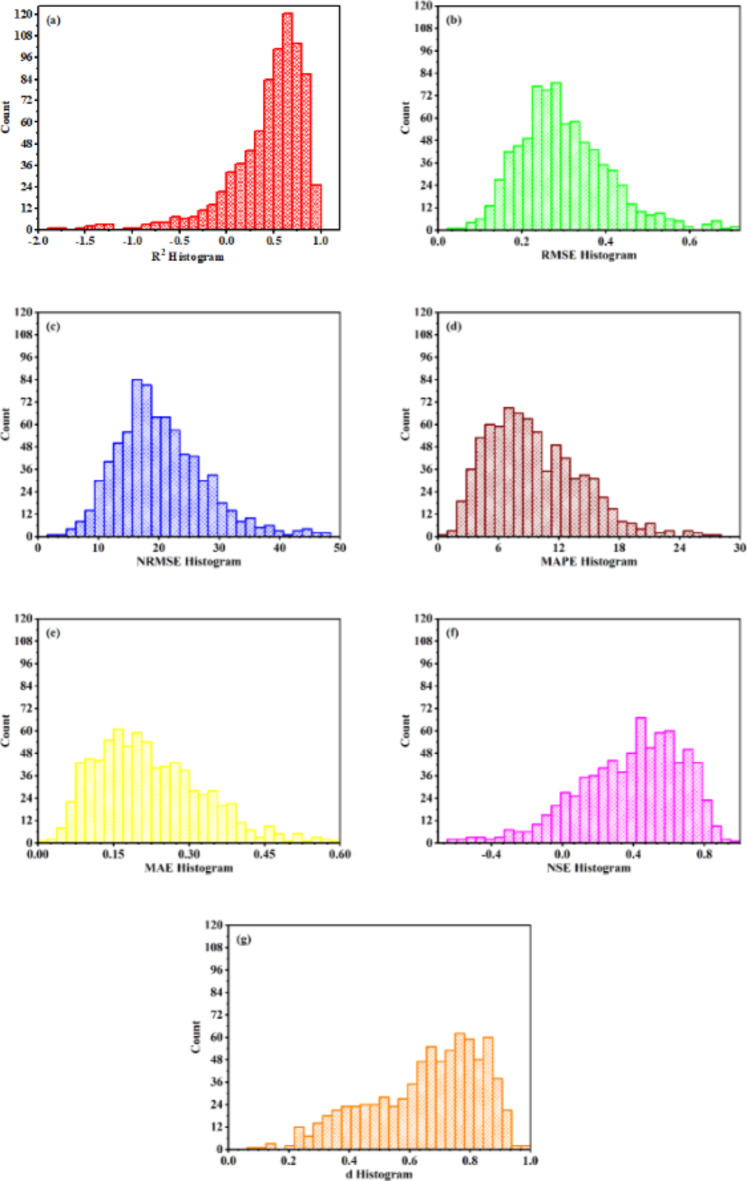
Assessment of model evaluation criteria for tool life trained ANN models (**a**) coefficient of determination R^2^ (**b**) root mean square error RMSE (**c**) normalized root mean square error NRMSE (**d**) mean absolute error MAE (**e**) mean absolute percentage error MAPE (**f**) Nash and Sutcliffe efficiency NSE (**g**) modified agreement index d.

The details of MLP-ANN models trained on special L_18_ (6^1^3^3^) orthogonal array experimental design are given in Table 4. All trained models for tool life assessed on model evaluation criteria are shown in Fig. 6. Total 4.3% of the models for tool life have reached the selection criteria with 0% from slab 1, 4% from slab 2, 1.6% from slab 3 and 4.96% from slab 4. The slabs are given in Table 3. The model configuration with 4 hidden layers and 6, 12, 6 and 12 neurons from first to fourth layer respectively, showed maximum performance in goodness of fit criteria. Among the models reaching selection criteria the model configuration with 4 hidden layers and 6, 10, 12 and 6 neurons from first to fourth layer respectively, showed minimum performance in goodness of fit criteria.

**Fig. 7:**
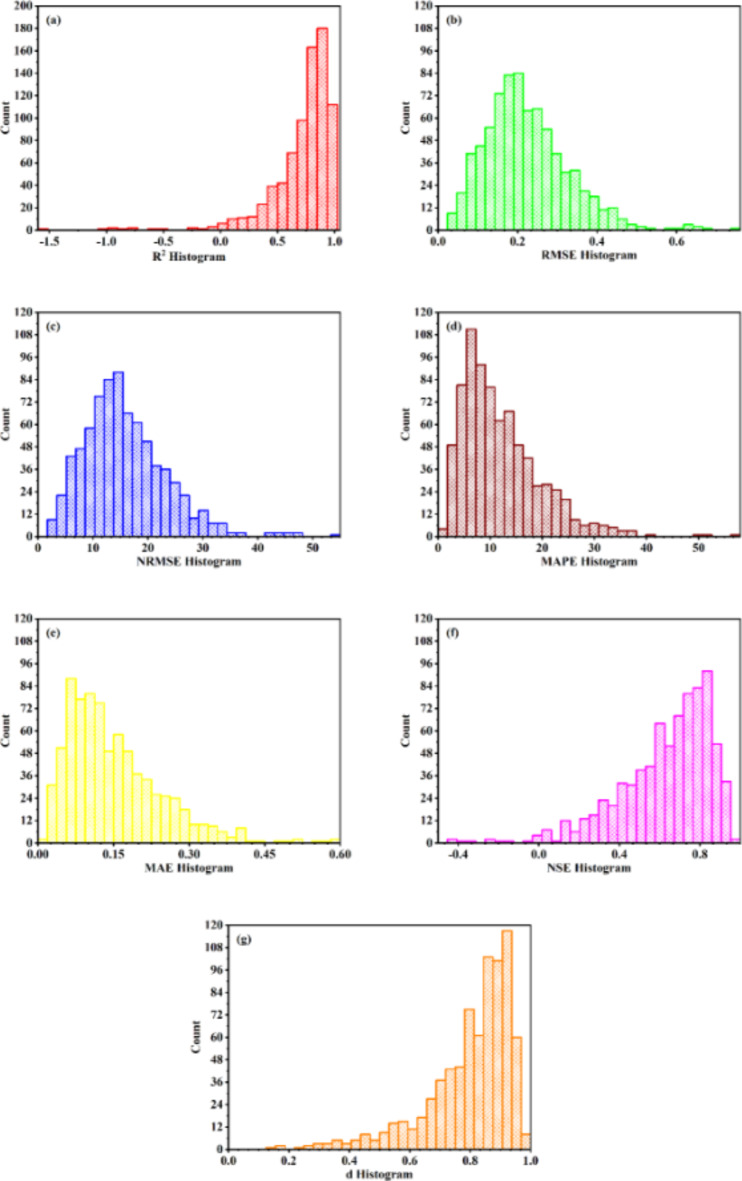
Assessment of model evaluation criteria for surface finish trained ANN models (**a**) coefficient of determination R^2^ (**b**) root mean square error RMSE (**c**) normalized root mean square error NRMSE (**d**) mean absolute error MAE (**e**) mean absolute percentage error MAPE (f) Nash and Sutcliffe efficiency NSE (**g**) modified agreement index d.

Similarly, all trained models for surface roughness assessed on model evaluation criteria are shown in Fig. 7. Total 24.35% of the models for surface roughness have reached the selection criteria with 40% from slab 1, 36% from slab 2, 25.6% from slab 3 and 23.52% from slab 4. The model configuration having two hidden layers with 6 neurons in first layer and 4 neurons in second layer, showed maximum performance in goodness of fit criteria. Among the models reaching selection criteria, the model configuration having 4 hidden layers with 12, 4, 4 and 4 neurons in layer one to four respectively, showed minimum performance in goodness of fit criteria. The MLP-ANN models are selected for further analysis i.e., the effect of higher machine learning model dimensions on the compromise made in reduced low resolution experimental design.

#### SVM

**Fig. 8:**
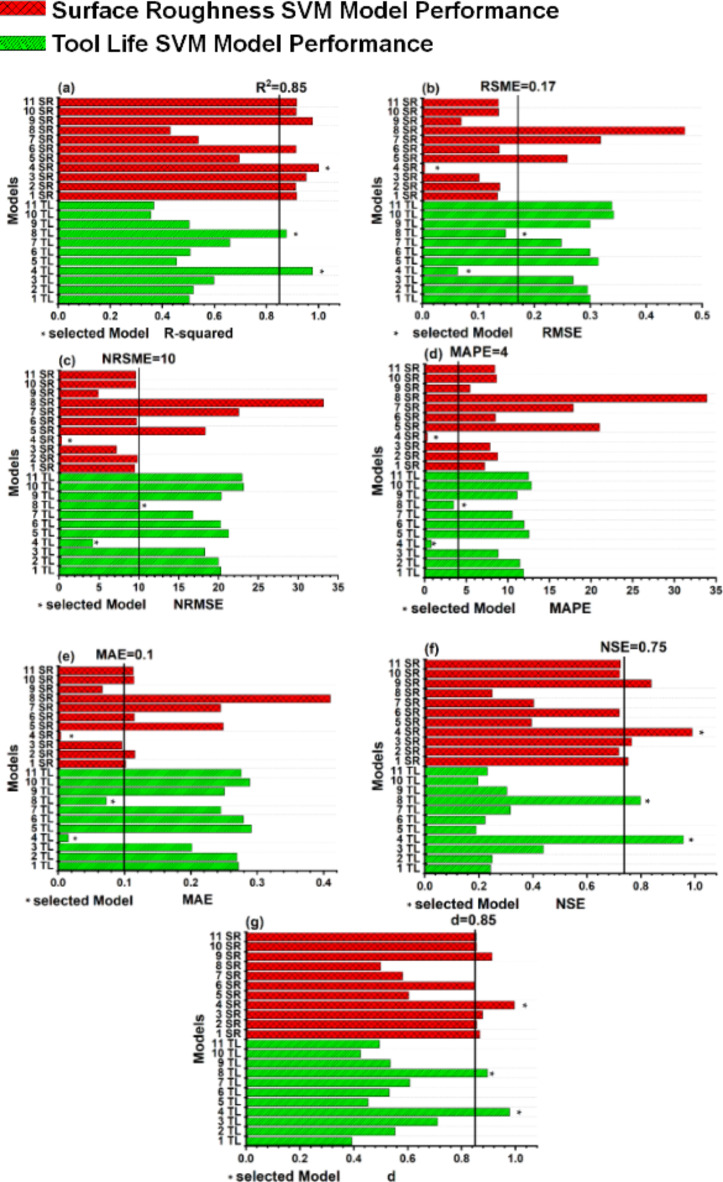
Assessment of model evaluation criteria for surface finish and tool life trained SVM models (**a**) coefficient of determination R^2^ (**b**) root mean square error RMSE (**c**) normalized root mean square error NRMSE (**d**) mean absolute error MAE (**e**) mean absolute percentage error MAPE (**f**) Nash and Sutcliffe efficiency NSE (**g**) modified agreement index d.

The SVM models given in Table 5 are analyzed using model evaluation criteria. Most of the models performed poorly as represented in Fig. 8. For each selection criteria number represents model number according to the Table 5 and “SR” and “TL” represents model trained for surface roughness and tool life respectively. The poor performance of the models might be attributed to low training data volume that is being fed to SVMs during training process with each data point orthogonal to second one. This can lead to a very high dimensional hyper-space with very low data volume making it difficult to understand input-output causal relationship. Despite very low data volume few algorithms performed significantly well in one or more than one selection criteria. However, to select a model for further analysis a strict criterion is followed i.e., a model needs to perform in all seven criteria and reach the set threshold value. The models reaching the selection criteria are presented with * sign in Fig. 8. SVM model number 4TL with Bayesian optimization and SVM model number 8TL with random search are selected for tool life. Both of these models are trained using cubic kernel function. SVM model 4SR with Bayesian optimization and cubic kernel function is selected for workpiece surface roughness. The models with linear, quadratic and Gaussian kernel functions failed to perform in one or several selection criteria.

#### Trees

**Fig. 9:**
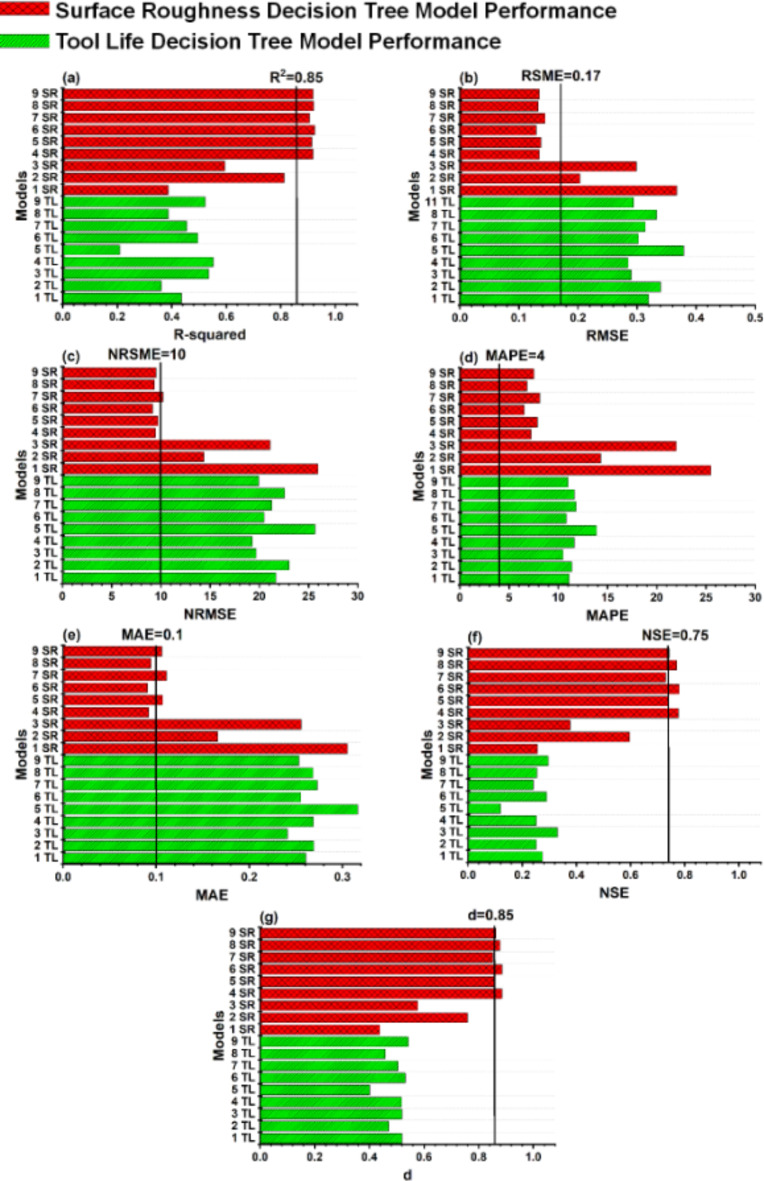
Assessment of model evaluation criteria for surface finish and tool life trained Tree models (**a**) coefficient of determination R^2^ (**b**) root mean square error RMSE (**c**) normalized root mean square error NRMSE (**d**) mean absolute error MAE (**e**) mean absolute percentage error MAPE (**f**) Nash and Sutcliffe efficiency NSE (**g**) modified agreement index d.

Figure 9 represents the goodness of fit for decision trees. Trees with various combinations and wide range of hyper-parameters are tested for both tool life and surface roughness training. The performance of trees is found to be very limited for the L^18^ (6^1^3^3^) orthogonal array low resolution data set under investigation and cannot reach the model selection criteria. Therefore, decision trees are also not selected for further analysis.

### Confirmation through external validation and MLP-ANN dimensional analysis

The AI models are finally evaluated on set of new experiments as given in Table 6. Experimental set given to AI models for prediction is unseen for the models and not introduced during training process. The best model is considered for response surface on the basis of percentage relative error and model robustness. The experimental results with particular setting for surface roughness and tool life are given in Table 7.

**Fig. 10:**
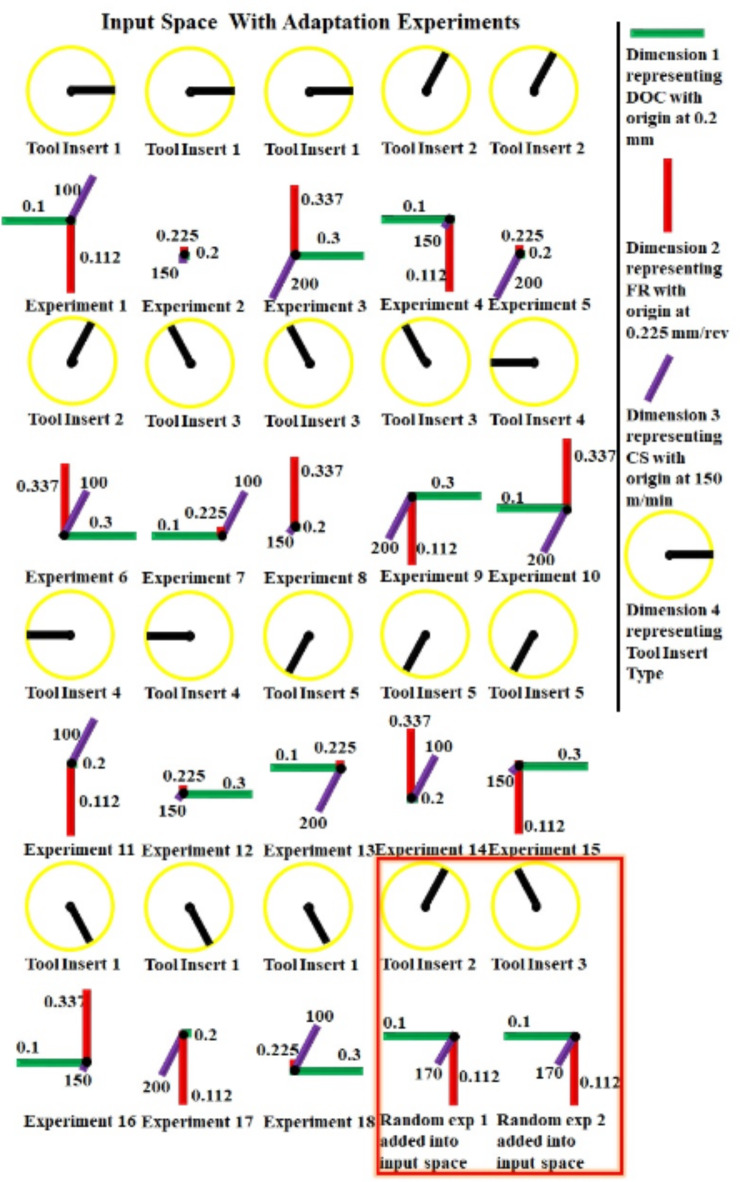
Input space for adaptive learning indicating all four experimental dimensions and two new added random experiments.

#### Surface roughness

**Fig. 11:**
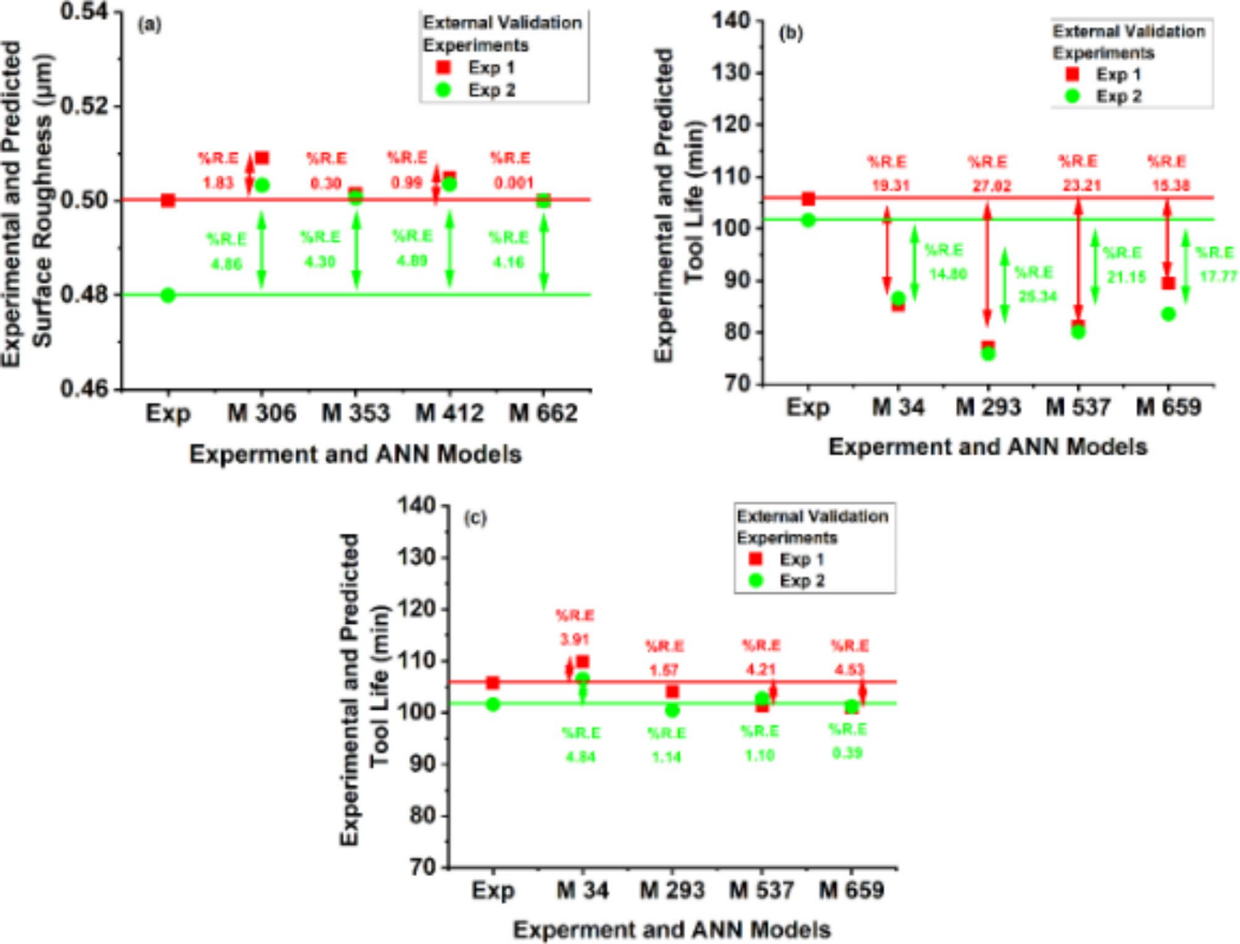
Percentage relative error of experimental and predicted values (**a**) Surface roughness (**b**) Tool life before adaptation (**c**) Tool life after adaptation.

The selected AI models trained for surface roughness are evaluated against experimental results. The AI models with less than 10% relative error in predicted values are taken as working models. The 10% relative error is selected to accommodate possible hidden errors that can occur during experimental process and AI models generalization. In MLP-ANN for surface roughness no model predicted significantly well in slab 1 and slab 2 (the slabs are given in Table 3). The slab 3 that is comprised of three hidden layers have 3 models successfully predicting the surface roughness of unseen experiments. Similarly, the slab 4 that is comprised of four hidden layers have 9 models successfully predicting the surface roughness of unseen experiments. A percentage relative error of less than 5% is achieved for 4 models having four hidden layers. It is also interesting to note that all models predicting surface roughness close to experimental values have greater than three times the number of neurons compared to inputs in hidden layer. The results suggest that with the increase in hidden layer depth and width the prediction of low resolution experiments can be improved. Figure 11a represents predictions of models (M) with less than 5% relative error for surface roughness. MLP-ANN model with less than 5% relative error is selected for making AI-response surfaces. The selected SVM models have failed to predict surface roughness values in the accepted range. Therefore, these models are not included further for making response surface through AI model. SVMs are very powerful models and needs to be considered in future for low resolution experimental design with high data density.

**Table 7:** Results of external validation experiments and details of new experiments added for adaptive learning.

No	Tool insert type	DOC (mm)	FR (mm/rev)	CS (m/min)	SR (µm)	TL (min)
Experiments used for models external validation						
1	TiCN (3)	0.1	0.112	200	0.5	105.774
2	TiN (2)	0.1	0.112	200	0.48	101.63
Experiments added in training data of tool life models for adaptive learning						
1	TiCN (3)	0.1	0.112	170	0.460	112.62
2	TiN (2)	0.1	0.112	170	0.483	76.87

#### Tool life & enhanced deep learning adaptive knowledge

The selected AI models trained for tool life are evaluated against experimental results. The AI models with less than 10% relative error in predicted values are taken as working models. All selected MLP-ANN models and SVM models have failed to predict unseen experimental tool life results with in the acceptable range. This issue is resolved by adaptive learning of AI models. AI is a very powerful tool and AI can change its behavior with every bit of new information provided to it. For adaptive learning two new random experiments as given in Fig. 10 are performed with in the input space of special L_18_ (6^1^3^3^) orthogonal array experimental design in the hope to successfully map the missing nonlinearities and aliased interaction in the knowledge learned and stored in the memory of the deep learning models. The details of experimental settings and corresponding results are given in Table 7. All selected models are trained using eighteen special L_18_ (6^1^3^3^) orthogonal array experimental design data points and two newly performed experimental data points. AI models are re-evaluated and tested for external validation experiments.

MLP-ANN models out performs SVM models for tool life and similar to surface roughness case SVM models are dropped for tool life as well for response surface analysis. The MLP-ANN models trained with 20 experimental data points having less than 5% relative error (Fig. 11c) are compared with same models trained with 18 experimental data points (Fig. 11b). The percentage relative error significantly reduced by just adding 2 new experimental data points into the Taguchi’s L_18_ (6^1^3^3^) orthogonal array training input space. Figure 11c also indicates the significance of higher dimensional space in AI models compensating low resolution experimental design. AI-Taguchi hybrid technique can be helpful in reduction of experimental cost. Moreover, the AI models ability to not rely on degree of freedom as in case of regression enable these models to predict complex input-output causal relationships without the restriction of minimum number of experimental data condition. However, the selection of AI model for prediction requires a carefully followed criteria. As in this study, few models achieving acceptable values for seven model performance criteria (i.e., R^2^, RMSE, NRMSE, MAE, MAPE, NSE and d) failed to predict on acceptable range on external validation data set.

#### AI-response surface for surface roughness

**Fig. 12:**
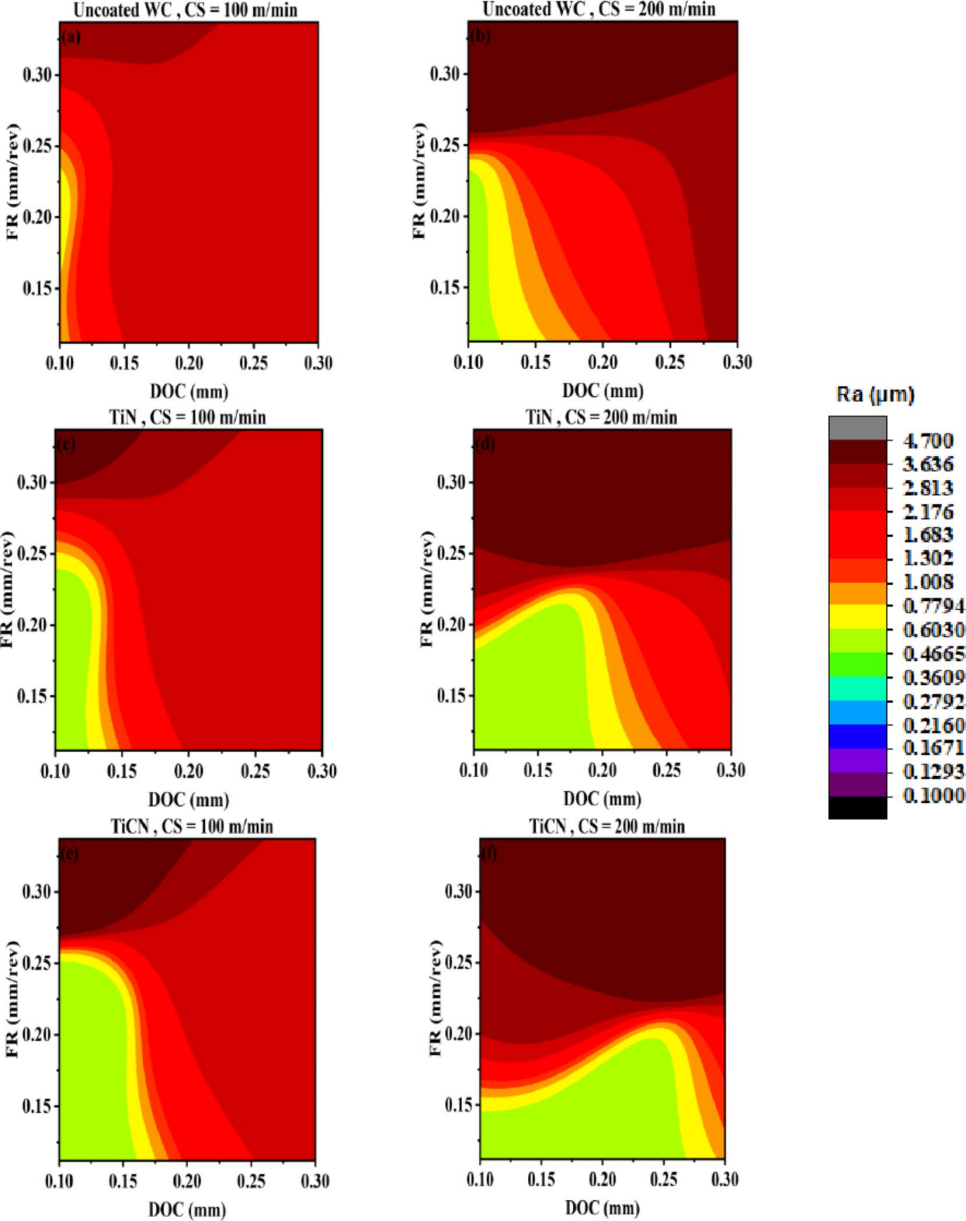

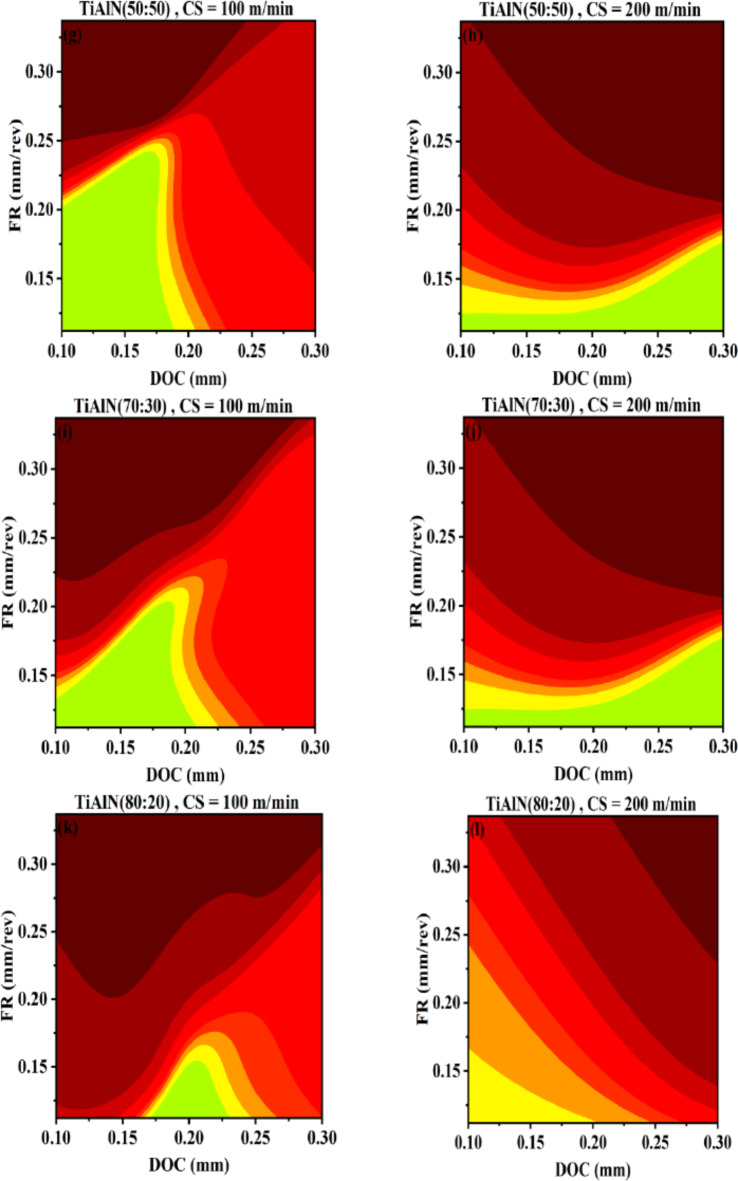
AI-response surface between DOC and FR for workpiece surface roughness (**a**) Uncoated WC, minimum CS (**b**) Uncoated WC, maximum CS (**c**) TiN/WC, minimum CS (**d**) TiN/WC, maximum CS (**e**) TiCN/WC, minimum CS (**f**) TiCN/WC, maximum CS (**g**) TiAlN(50:50)/WC, minimum CS (**h**) TiAlN(50:50)/WC, maximum CS (**i**) TiAlN(70:30)/WC, minimum CS (**j**) TiAlN(70:30)/WC, maximum CS (**k**) TiAlN(80:20)/WC, minimum CS (**l**) TiAlN(80:20)/WC, maximum CS.

**Fig. 13:**
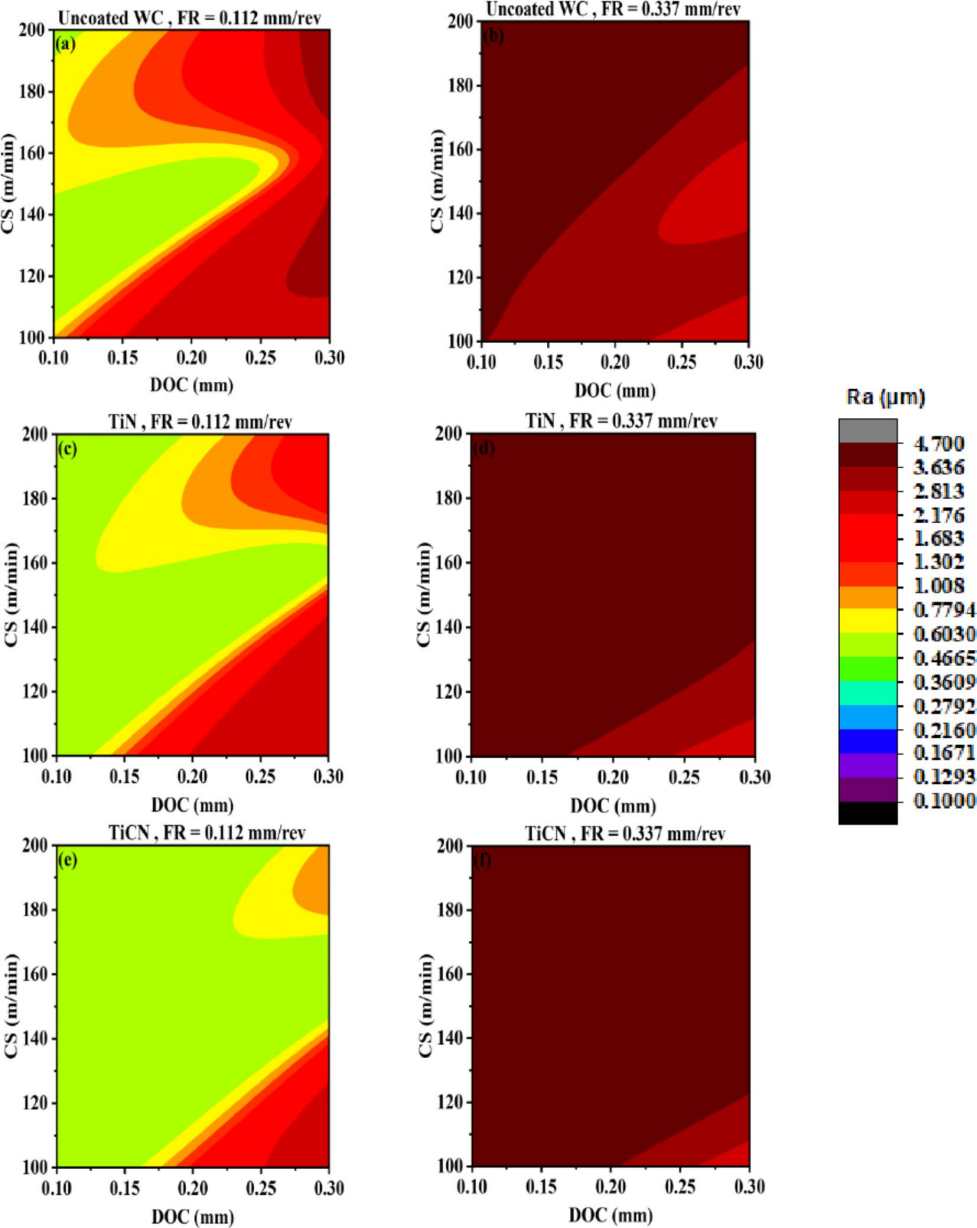

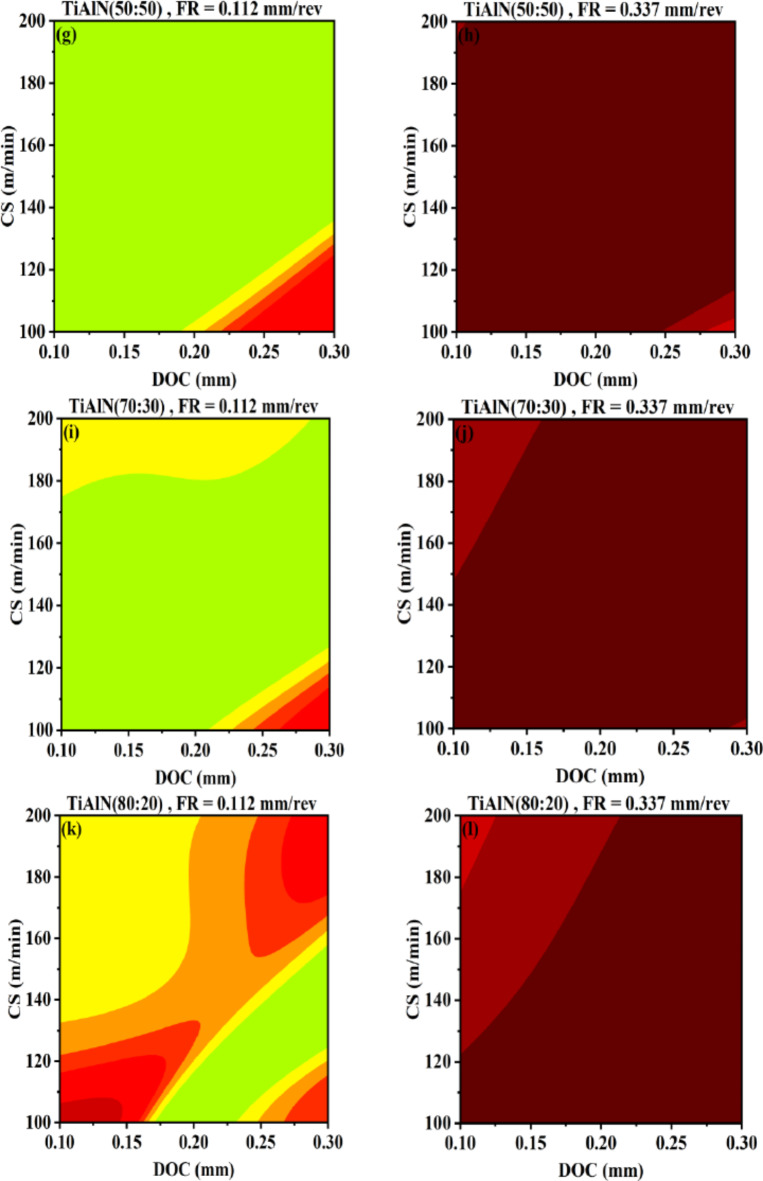
AI-response surface between DOC and CS for workpiece surface roughness (**a**) Uncoated WC, minimum FR (**b**) Uncoated WC, maximum FR (**c**) TiN/WC, minimum FR (**d**) TiN/WC, maximum FR (**e**) TiCN/WC, minimum FR (**f**) TiCN/WC, maximum FR (**g)** TiAlN(50:50)/WC, minimum FR (**h**) TiAlN(50:50)/WC, maximum FR (**i**) TiAlN(70:30)/WC, minimum FR (**j**) TiAlN(70:30)/WC, maximum FR (**k**) TiAlN(80:20)/WC, minimum FR (**l**) TiAlN(80:20)/WC, maximum FR.

**Fig. 14:**
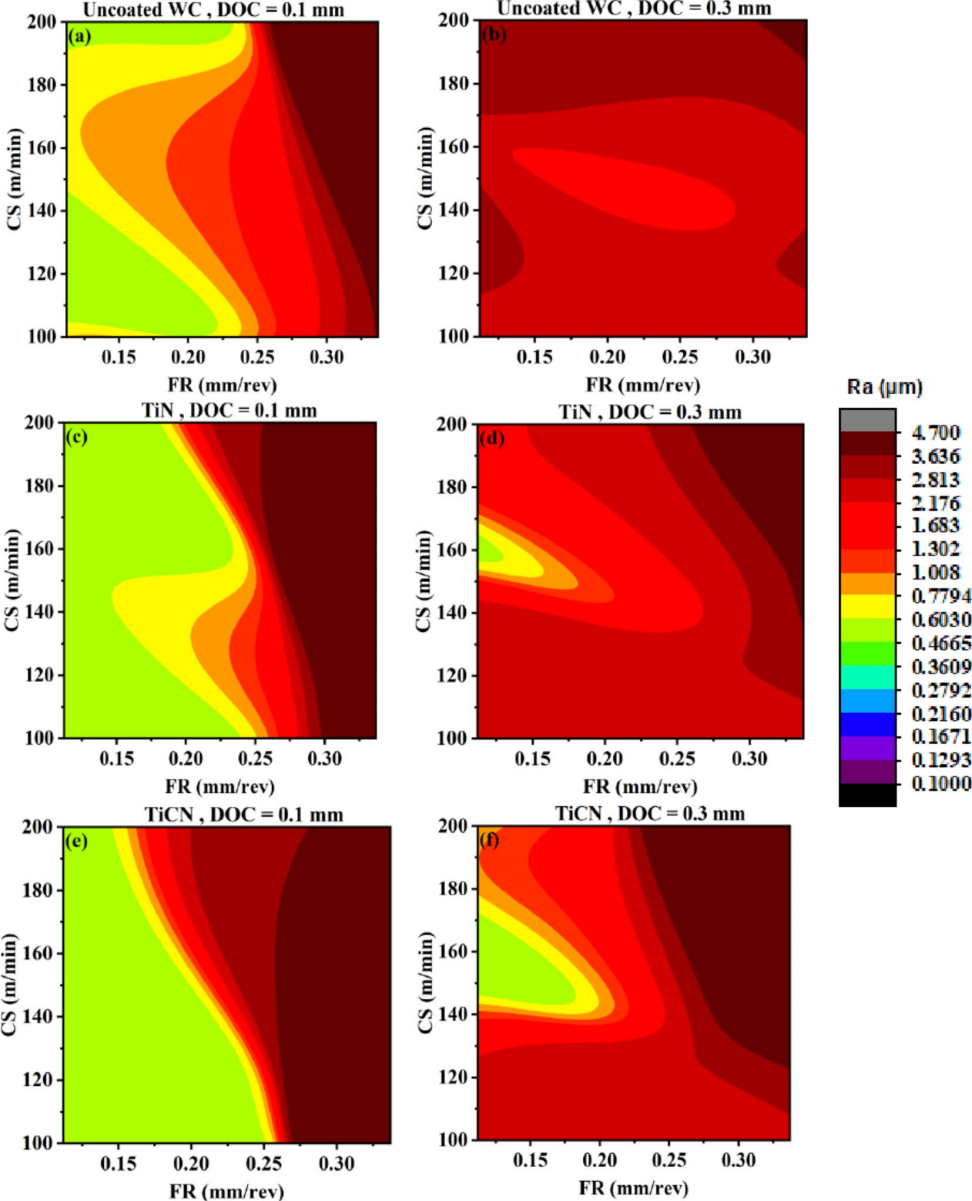

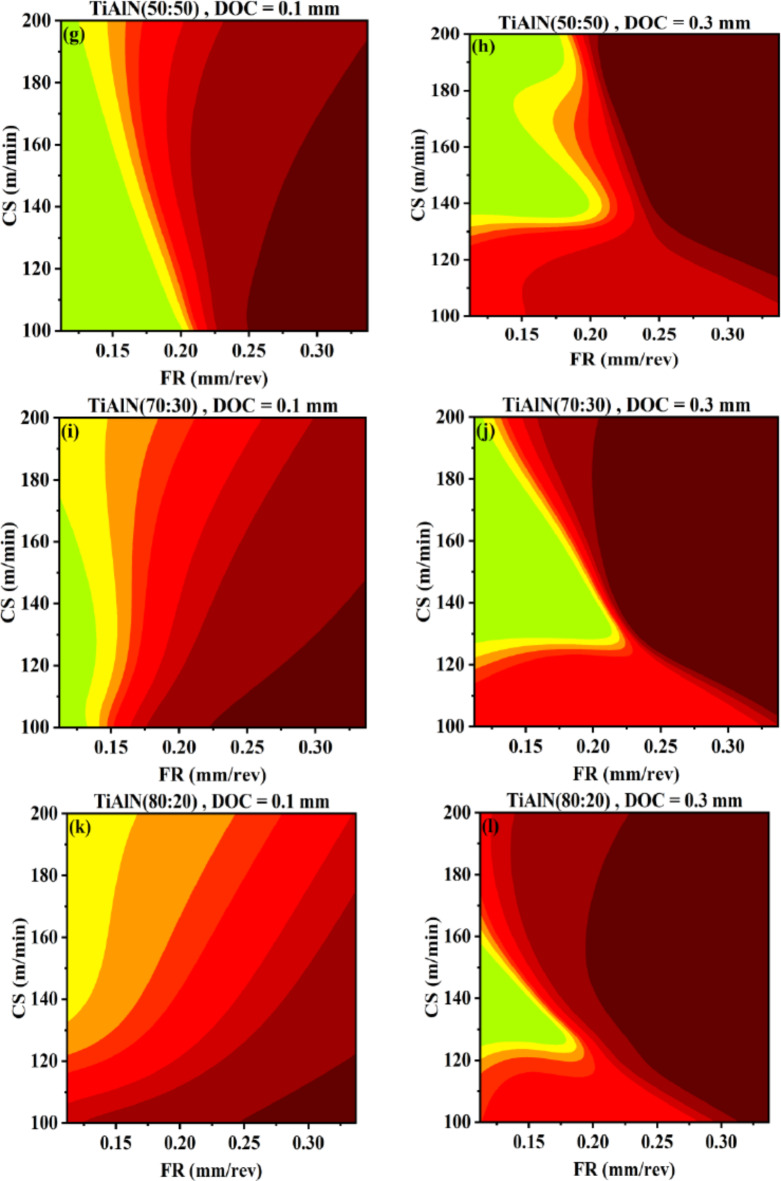
AI-response surface between FR and CS for workpiece surface roughness (**a**) Uncoated WC, minimum DOC (**b**) Uncoated WC, maximum DOC (**c**) TiN/WC, minimum DOC (**d**) TiN/WC, maximum DOC (**e**) TiCN/WC, minimum DOC (**f**) TiCN/WC, maximum DOC (**g**) TiAlN(50:50)/WC, minimum DOC (**h**) TiAlN(50:50)/WC, maximum DOC (**i**) TiAlN(70:30)/WC, minimum DOC (**j**) TiAlN(70:30)/WC, maximum DOC (**k**) TiAlN(80:20)/WC, minimum DOC (**l**) TiAlN(80:20)/WC, maximum DOC.

The artificial intelligence based response surfaces are created for surface roughness. This tool can help in visualization of input-output relational space with interaction and range information. Figures 12, 13 and 14 are used to identify suitable range of input parameters for the analysis of tool life on the basis of lower surface roughness range. The machining is performed on AA 7075 that is aerospace grade aluminum alloy. In aerospace industry the quality of the product is very critical and tool life can be compromised for achieving higher quality, if achieving higher tool life means poor surface finish. However, a solution with higher surface finish (always first preference) and higher tool life is desirable^60,61^. Therefore, ranges are identified for high workpiece surface finish or low workpiece surface roughness and tool life is analyzed with in those ranges.

Figure 12 represents AI-response surfaces of all six tool inserts. The effect of changing DOC and FR is studied by holding CS at minimum and maximum level. The regions are separated between minimum and maximum surface roughness values. The legend given is showing natural log separation scheme between separated regions. AI-response surfaces confirmed that a non-linear and interactive relation exists for DOC and FR corresponding to surface roughness. The interactive nature of DOC and FR has also reported by the research community^17,62^. At higher cutting speed, range of DOC is increased with reduction in FR range for obtaining lower surface roughness. This is true for all tool inserts except TiAlN(80:20) Fig. 12a−l. Travelling diagonally on AI-response surfaces indicates significance of FR at lower cutting speed and DOC at higher cutting speed. The higher influence of DOC at higher cutting speed to surface roughness has been also observed for different tool insert and workpiece^62^. The coated tool inserts perform significantly better compared to uncoated WC except for TiAlN(80:20). For high DOC, high feed rate and at maximum cutting speed poor surface roughness is achieved. This result is also confirmed by Fig. 13.

Figure 13 represents AI-response surfaces of all six tool inserts relating DOC and CS with surface roughness. A nonlinear relation and interactive behavior is observed for DOC and CS corresponding to surface roughness^17,63^. The FR is held at maximum and minimum experimental values. For uncoated WC, TiN and TiAlN(80:20) coated WC tool inserts, highly interactive relation exist between DOC and CS at low feed rate. A reduced interactive effect of DOC and CS on output is obtained for TiCN, TiAlN(50:50) and TiAlN(70:30). Figure 13 suggest that a full range of CS needs to be tested for tool life as minimum surface roughness can be achieved in full range keeping other parameters at suitable settings. Figure 13k indicates that for TiAlN(80:20) tool life model the suitable CS range to evaluate tool life is between 100 and 155 with DOC between 0.17 and 0.3. Outside this range the surface roughness is high and if higher tool life is achieved that will be on the expense of surface finish. Comparatively non-linear output response with the change in both DOC and CS vanishes at higher feed rate holding value as can be seen in Fig. 13b,d,f,h,l. This again confirms that the combination with maximum FR, DOC and CS needs to be avoided for better production quality output^64,65^.

 Figure 14 represents AI-response surfaces of all six tool inserts relating FR and CS with surface roughness. The DOC is held at maximum and minimum experimental values. A non-linear and interactive relation exist between FR and cutting speed with respect to workpiece surface roughness^66,67^. Figure 14 suggest that the FR is comparatively more significant parameter than cutting speed in terms of producing change in surface roughness. The result is in conformance with previously published study by Akgün et al., indicating feed rate as most significant variable influencing surface roughness compared to DOC and CS^66^. Akthar et al., also designates the first rank to feed rate for variable influencing surface roughness compared to DOC and CS^63^. Figure 14a, b indicates that at higher DOC both FR and cutting speed have worsen effect on surface roughness compared to low DOC setting. Similar behavior is observed for TiN, TiCN and TiAlN(50:50) coated WC tool inserts Fig. 14b–g. TiAlN(70:30) and TiAlN (80:20) coated tool inserts benefits from the interactive relation between FR and CS and higher DOC setting as given in Fig. 14i,l.

The ranges of DOC, FR and CS for each tool insert are given in Table 8. The ranges are selected on the basis of AI-response surfaces given in Figs. 12, 13 and 14. Tool life is evaluated in the given ranges for respective tool inserts.

**Table 8:** Range selection for tool life evaluation based on AI-Response surface for surface roughness.

Tool insert type	DOC (mm)	FR (mm/rev)	CS (m/min)
1	0.1–0.25	0.112–0.225	100–200
2	0.1–0.3	0.112–0.24	100–200
3	0.1–0.3	0.112–0.25	100–200
4	0.1–0.3	0.112–0.24	100–200
5	0.1–0.3	0.112–0.225	100–200
6	0.17–0.3	0.112–0.18	100–155

### AI-response surface for tool life

**Fig. 15:**
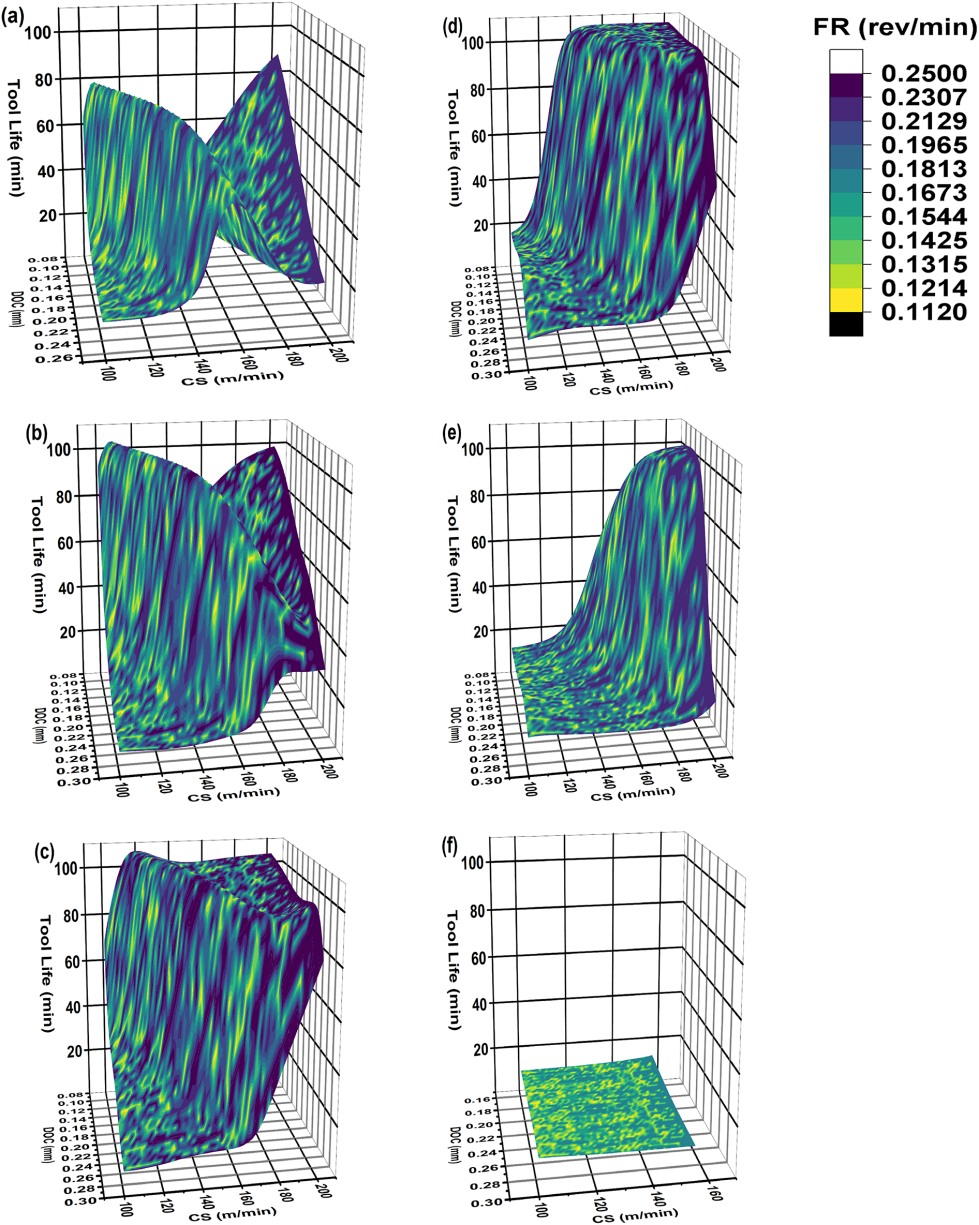
The 3D AI-response surface between DOC, FR and CS for tool life (**a**) Uncoated WC (**b**) TiN/WC (**c**) TiCN/WC (**d**) TiAlN(50:50)/WC (**e**) TiAlN(70:30)/WC (**f**) TiAlN(80:20)/WC.

The AI-response surfaces for tool life and all tool inserts are given in Fig. 15. The AI-response surfaces are made without holding any operational parameter and the ranges mentioned in Table 8 are simulated through adaptive learning enhanced MLP-ANN model. The DOC and CS are presented on x-axis and z-axis respectively. The FR is given as dark and light color region variations on surface profile. The regions are separated between minimum and maximum FR values. The legend given is showing natural log separation scheme between different regions. The tool life i.e., measured response to changing operational parameters is given on y-axis. This representation is given for ease in readability of given AI-response surface. It is interesting to note that the general behavior of uncoated WC, TiN coated WC and TiCN coated WC is quite similar Fig. 15a–c. The in-house developed novel alloy based coated WC tool inserts show different behavior compared to above mention three tool inserts. The general behavior of TiAlN(50:50) coated WC and TiAlN(70:30) coated WC is quite comparable as given in Fig. 15d,e. Minimum tool life is obtained for TiAlN(80:20) as given in Fig. 15f.

The AI-response surface for uncoated WC tool insert is given in Fig. 15a. The uncoated WC showed very low tool life and mostly remained under 80 min except for the operational parameters combination of minimum DOC, maximum FR and CS. However, at this combination the surface roughness is quite high as can be seen in Fig. 12b.

The AI-response surfaces for TiN coated WC and TiCN coated WC tool inserts are presented in Fig. 15b,c respectively. Both tool inserts showed high tool life especially with the increase in DOC and CS along with wide range of FR. Similar tool wear behavior with respect to increase in DOC and CS can be observed in previous reseach work^68,69^. The ranges where tool life is high also have minimum surface roughness therefore, multiple operational parameter settings can be selected for achieving particular goal under certain machining condition.

The AI-response surfaces for TiAlN(50:50) coated WC and TiAlN(70:30) coated WC tool inserts are presented in Fig. 15d,e respectively. The results indicate that TiAlN(50:50) outperform perform TiAlN(70:30) in terms of obtaining higher tool life. TiAlN(50:50) coated WC tool inserts only performs on lower DOC and shorter CS (high CS and low CS sweep) range compared to TiN coated WC and TiCN coated WC tool inserts. This DOC and CS range is further reduced for TiAlN(70:30).

The systematic decision making process for finding best solution preference order can be summarized as obtaining an AI model that can represent the physical system (Sections “Tool life and surface roughness”, “Model evaluation and selection”, “Confirmation through external validation and MLP-ANNdimensional analysis”), identification of ranges for high workpiece surface finish or low workpiece surface roughness (Figs. 12, 13 and 14, range summary given in Table 8) and evaluating tool life for identified ranges (Fig. 15). The analysis of Figs. 12, 13, 14 and 15 indicates that by considering surface roughness as primary decision criterion and tool life as secondary; the order of tool inserts on the basis of performance is TiN coated WC > TiCN coated WC > TiAlN(50:50) coated WC > TiAlN(70:30) coated WC > uncoated WC > TiAlN(80:20) coated WC.

The results suggest that AI-Taguchi hybrid approach can be utilized for the modelling and mining lost information (nonlinearity and aliasing) due to low resolution orthogonal array experimental design. Usually, Taguchi orthogonal arrays are used to evaluate the main effects with respect to a response and screening of input variables. After that if causal relationship and interaction information is required then a full factorial experimental design is usually implemented on critical input variables identified during Taguchi screening process. The machining data discussed in this study conventionally would require 18 experiments for Taguchi’s L_18_ (6^1^3^3^) orthogonal array and minimum 9 experiments for making full factorial design of best identified tool insert and two operational parameters with three levels. This will lead to a total of 27 experiments required to model the causal relationship and develop the optimized manufacturing process. In this study a total of 20 experiments (18 experiments for Taguchi’s L_18_ (6^1^3^3^) orthogonal array for AI model training and 2 random experiments within the Taguchi L_18_ (6^1^3^3^) orthogonal array input space are conducted (for AI model enhancement through adaptive learning) to achieve effective AI process models and AI-response surface based optimized process development. This suggest that the AI-Taguchi hybrid approach requires 26% less experiments compared to conventional Taguchi based technique. This saving in experimental cost may further increase for industrial process development problems involving higher number of input variables or levels. The authors encourage research and industrial communities to work on higher dimensional problems with AI-Taguchi hybrid technique to further investigate its experimental cost saving potential in manufacturing process development and industrial problem solving.

## Conclusion and future work

The artificial intelligence models are trained for Taguchi’s L_18_ (6^1^3^3^) orthogonal array experimental design. The experimental data of dry finishing turning of AA7075 using six different tool inserts is used to train the AI models. The models are selected on seven goodness of fit criteria along with external validation. AI-response surfaces are made to understand the relations present. Following are the key conclusions of this study:

- The method applied for the analysis that includes Taguchi orthogonal array based experimentation, artificial intelligence based model training, implementation of model selection criteria, AI model enhancement by adaptive learning and finding AI-response surface based optimized solution for specific requirement can be utilized to save experimental cost and better decision making in wide range of industries like automotive, manufacturing and aerospace etc.
- The reduced experimental dimension in low resolution experimental design can be compensated with adaptive learning enhanced AI models based on randomly selected experiments within the input space and significant savings can be achieved in terms of cost and time for effective machining process optimization.
- AI models are not dependent on degree of freedom available in an experimental design as opposed to conventional regression based process optimization techniques.
- AI-Taguchi models can be successfully implemented for obtaining causal relationships between inputs and outputs with reduced number of experiments saving both time and experimental cost. This can ensure product development with high confidence and low cost. The experimental savings recorded in this study for four input variables are approximately 26% as opposed to conventional Taguchi analysis followed by critical variable full factorial design and response surface optimization approach.
- This experimental savings can be increased if number of observed input parameters and corresponding levels are high. The authors encourage research and industrial communities to work on higher dimensional problems with AI-Taguchi hybrid technique to confirm AI-Taguchi utility in manufacturing process development and industrial problem solving.
- MLP-ANN models performed significantly well in both training and external validation for workpiece surface roughness and tool life predictions. SVM models have failed in external validation process whereas, decision trees showed very low capabilities in learning problem behavior for low resolution input space.
- The AI-response surface suggests that the combination of high DOC, high feed rate and maximum cutting speed needs to be avoided as it can result in poor workpiece surface finish.
- Novel alloy based coated tool inserts showed different behavior compared to non-alloy based coated tool inserts. This property is identified by AI-response surface based on MLP-ANN model for tool life.
- TiN coated WC, TiCN coated WC, TiAlN(50:50) coated WC and TiAlN(70:30) coated WC tool inserts showed higher DOC range approximately 25% higher as compared to uncoated WC tool insert and 35% higher as compared to TiAlN(80:20) coated WC tool inserts for obtaining greater surface finish. TiAlN(80:20) coated WC tool insert showed ~ 45% lower cutting speed range for obtaining greater surface finish as compared to all other studied tool inserts (refer to Table 8).
- Both TiN coated WC and TiAlN(50:50) coated WC tool inserts showed ~ 11% and ~ 46% higher feed rate range for obtaining greater surface finish as compared to uncoated WC and TiAlN(80:20) coated WC tool inserts respectively. Similarly, TiCN coated WC tool insert showed ~ 18% and ~ 50% higher feed rate range for obtaining higher surface finish as compared to uncoated WC and TiAlN(80:20) coated WC tool inserts respectively (refer to Table 8).
- The maximum tool life obtained for TiN coated WC and TiCN coated WC tool inserts is approximately 13% higher as compared to maximum tool life obtained for uncoated WC tool insert and approximately 84% higher as compared to maximum tool life obtained for TiAlN(80:20) coated WC tool insert (refer to Fig. 15).
- TiN coated WC > TiCN coated WC > TiAlN(50:50) coated WC > TiAlN(70:30) coated WC > uncoated WC > TiAlN(80:20) coated WC is the tool inserts performance order for operational parameters studied ranges of AA7075 dry finish turning operation.

## Data Availability

The readers can request the corresponding author for the data underlying the findings of the study.

## Abbreviations

DOC: Depth of cut
SVMs: Support vector machines
FR: Feed rate
SR: Surface roughness
CS: Cutting speed
TL: Tool Life
MLP-ANNs: Multi-layer perceptron based artificial neural networks

## References

[1] Kammer, C. *Aluminum and Aluminum Alloys* (Springer, 2018).

[2] Rana, R., Purohit, R. & Das, S. Reviews on the influences of alloying elements on the microstructure and mechanical properties of aluminum alloys and aluminum alloy composites. *Int. J. Sci. Res. Publi.* **2**(6), 1–7 (2012).

[3] Zhang, Y. et al. Corrosion of aluminum alloy 7075 induced by marine aspergillus terreus with continued organic carbon starvation. *Npj Mater. Degrad.* **6**(1), 27 (2022).

[4] Zhou, B. et al. Microstructure evolution of recycled 7075 aluminum alloy and its mechanical and corrosion properties. *J. Alloys Compd.* **879**, 160407 (2021).

[5] Cerchier, P. et al. PEO coating containing copper: a promising anticorrosive and antifouling coating for seawater application of AA 7075. *Surf. Coat. Technol.* **393**, 125774 (2020).

[6] Andreatta, F., Terryn, H. & De Wit, J. Corrosion behaviour of different tempers of AA7075 aluminium alloy. *Electrochim. Acta* **49**(17–18), 2851–2862 (2004).

[7] Karabay, S., Bayraklılar, M. & Balcı, E. Influence of different heat treatments on the solid particle erosion behavior of aluminum alloy AA 7075 in industrial applications. *Acta Phys. Pol. A* **127**(4), 1052–1054 (2015).

[8] Ramkumar, K. et al. Investigations on microstructure, mechanical, and tribological behaviour of AA 7075-x wt.% TiC composites for aerospace applications. *Arch. Civil Mech. Eng.* **19**, 428–438 (2019).

[9] Gupta, M. K. et al. Cutting forces and temperature measurements in cryogenic assisted turning of AA2024-T351 alloy: An experimentally validated simulation approach. *Measurement* **188**, 110594 (2022).

[10] Sahoo, S. P. & Datta, S. Dry machining performance of AA7075-T6 alloy using uncoated carbide and MT-CVD TiCN-Al2O3-coated carbide inserts. *Arab. J. Sci. Eng.* **45**(11), 9777–9791 (2020).

[11] Sreejith, P. & Ngoi, B. K. A. Dry machining: machining of the future. *J. Mater. Process. Technol.* **101**(1–3), 287–291 (2000).

[12] Shareef, I., Natarajan, M. & Ajayi, O. O. Dry machinability of aluminum alloys. In *World Tribology Congress* (2005).

[13] Singh, J. et al. State of the art review on the sustainable dry machining of advanced materials for multifaceted engineering applications: progressive advancements and directions for future prospects. *Mater. Res. Express* **9**(6), 064003 (2022).

[14] Gupta, M. K. et al. Hybrid cooling-lubrication strategies to improve surface topography and tool wear in sustainable turning of Al 7075-T6 alloy. *Int. J. Adv. Manuf. Technol.* **101**, 55–69 (2019).

[15] Sankaranarayanan, R. & Krolczyk, G. A comprehensive review on research developments of vegetable-oil based cutting fluids for sustainable machining challenges. *J. Manuf. Process.* **67**, 286–313 (2021).

[16] Pattnaik, S. K. et al. Dry machining of aluminum for proper selection of cutting tool: tool performance and tool wear. *Int. J. Adv. Manuf. Technol.* **98**, 55–65 (2018).

[17] Uddin, G. M. et al. Comparative performance analysis of cemented carbide, TiN, TiAlN, and PCD coated inserts in dry machining of Al 2024 alloy. *Int. J. Adv. Manuf. Technol.* **112**, 1461–1481 (2021).

[18] Kumar, C. S. et al. Applicability of DLC and WC/C low friction coatings on Al2O3/TiCN mixed ceramic cutting tools for dry machining of hardened 52100 steel. *Ceram. Int.* **46**(8), 11889–11897 (2020).

[19] Aizawa, T. et al. Self-lubrication mechanism via the in situ formed lubricious oxide tribofilm. *Wear* **259**(1–6), 708–718 (2005).

[20] Kara, F. et al. Effect of machinability, microstructure and hardness of deep cryogenic treatment in hard turning of AISI D2 steel with ceramic cutting. *J. Mater. Res. Technol.* **9**(1), 969–983 (2020).

[21] Marousi, M. et al. Initial tool wear and process monitoring during titanium metal matrix composite machining (TiMMC). *J. Manuf. Process.* **86**, 208–220 (2023).

[22] Cavaleiro, D. et al. Machining performance of TiSiN (Ag) coated tools during dry turning of TiAl6V4 aerospace alloy. *Ceram. Int.* **47**(8), 11799–11806 (2021).

[23] Iqbal, A. et al. Sustainable machining: Tool life criterion based on work surface quality. *Processes* **10**(6), 1087 (2022).

[24] Das, S. R., Dhupal, D. & Kumar, A. Study of surface roughness and flank wear in hard turning of AISI 4140 steel with coated ceramic inserts. *J. Mech. Sci. Technol.* **29**, 4329–4340 (2015).

[25] Padhan, S. et al. Sustainability assessment and machinability investigation of austenitic stainless steel in finish turning with advanced ultra-hard SiAlON ceramic tool under different cutting environments. *Silicon* **13**, 119–147 (2021).

[26] Bordin, A., Bruschi, S. & Ghiotti, A. The effect of cutting speed and feed rate on the surface integrity in dry turning of CoCrMo alloy. *Procedia Cirp* **13**, 219–224 (2014).

[27] Thomas, M. & Beauchamp, Y. Statistical investigation of modal parameters of cutting tools in dry turning. *Int. J. Mach. Tools Manuf.* **43**(11), 1093–1106 (2003).

[28] Magalhães, L. C. et al. Tool wear effect on surface integrity in AISI 1045 steel dry turning. *Materials* **15**(6), 2031 (2022). 10.3390/ma15062031 PMC8949504 35329482

[29] Dureja, J. et al. A review of empirical modeling techniques to optimize machining parameters for hard turning applications. *Proc. Inst. Mech. Eng. Part. B* **230**(3), 389–404 (2016).

[30] Ross, P. J. *Taguchi Techniques for Quality Engineering: Loss Function, Orthogonal Experiments, Parameter and Tolerance Design* (McGRAW-HILL, 1988).

[31] SK, T., Shankar, S. & K, D. Tool wear prediction in hard turning of EN8 steel using cutting force and surface roughness with artificial neural network. *Proc. Inst. Mech. Eng. C* **234**(1), 329–342. (2020).

[32] Boga, C. & Koroglu, T. Proper estimation of surface roughness using hybrid intelligence based on artificial neural network and genetic algorithm. *J. Manuf. Process.* **70**, 560–569 (2021).

[33] Zubair, S. W. H. et al. Dry finishing turning of AA7075 with binary and ternary nitrides and carbides ceramic-coated tools. *Int. J. Adv. Manuf. Technol.* **129**(1), 65–87 (2023).

[34] Imbrogno, S., Rotella, G. & Rinaldi, S. Surface and subsurface modifications of AA7075-T6 induced by dry and cryogenic high speed machining. *Int. J. Adv. Manuf. Technol.* **107**, 905–918 (2020).

[35] Pugazhenthi, A. et al. Predicting the effect of machining parameters on turning characteristics of AA7075/TiB 2 in situ aluminum matrix composites using empirical relationships. *J. Braz. Soc. Mech. Sci. Eng.* **40**, 1–15 (2018).

[36] Bhushan, R. K. Effect of tool wear on surface roughness in machining of AA7075/10áwt.% SiC composite. *Compos. Part. C: Open. Access.* **8**, 100254 (2022).

[37] Bull, S., Bhat, D. & Staia, M. Properties and performance of commercial TiCN coatings. Part 1: Coating architecture and hardness modelling. *Surf. Coat. Technol.* **163**, 499–506 (2003).

[38] Adesina, A. Y. et al. Electrochemical evaluation of the corrosion protectiveness and porosity of vacuum annealed CrAlN and TiAlN cathodic arc physical vapor deposited coatings. *Mater. Corros.* **70**(9), 1601–1616 (2019).

[39] Groover, M. P. *Fundamentals of Modern Manufacturing: Materials, Processes, and Systems* (Wiley, 2010).

[40] Ojolo, S. J. & Ogunkomaiya, O. A study of effects of machining parameters on tool life. *Int. J. Mater. Sci. Appl.* **3**(5), 183–199 (2014).

[41] Sreejith, P. Machining of 6061 aluminium alloy with MQL, dry and flooded lubricant conditions. *Mater. Lett.* **62**(2), 276–278 (2008).

[42] Stachowiak, G. & Batchelor, A. W. *Engineering Tribology* (Butterworth-Heinemann, 2013).

[43] Unal, R. & Dean, E. B. Taguchi approach to design optimization for quality and cost: An overview. In *1991 Annual Conference of the International Society of Parametric Analysts* (1990).

[44] Athreya, S. & Venkatesh, Y. Application of Taguchi method for optimization of process parameters in improving the surface roughness of lathe facing operation. *Int. Refereed J. Eng. Sci.* **1**(3), 13–19 (2012).

[45] Hamzaçebi, C. et al. *Taguchi Method as a Robust Design tool, in Quality Control - Intelligent Manufacturing, Robust Design and Charts* (intechopen, 2020).

[46] Awty-Carroll, D. et al. Using a Taguchi DOE to investigate factors and interactions affecting germination in Miscanthus sinensis. *Sci. Rep.* **10**(1), 1602 (2020). 32005862 10.1038/s41598-020-58322-x PMC6994594

[47] Abiyev, R., Mamedov, F. & Al-shanableh, T. Nonlinear neuro-fuzzy network for channel equalization. *Anal. Des. Intell. Syst. Using Soft Comput. Tech.* 327–336 (2007).

[48] Uddin, G. M. et al. Monte Carlo study of the high temperature hydrogen cleaning process of 6H-silicon carbide for subsequent growth of nano scale metal oxide films. *Int. J. Nanomanuf.* **9**(5–6), 407–430 (2013).

[49] Krzywanski, J. & Nowak, W. Artificial intelligence treatment of SO2 emissions from CFBC in air and oxygen-enriched conditions. *J. Energy Eng.* **142**(1), 04015017 (2016).

[50] Yıldız, Z. et al. Application of artificial neural networks to co-combustion of hazelnut husk–lignite coal blends. *Bioresour. Technol.* **200**, 42–47 (2016). 26476163 10.1016/j.biortech.2015.09.114

[51] Rajabizadeh, M. & Rezghi, M. A comparative study on image-based snake identification using machine learning. *Sci. Rep.* **11**(1), 19142 (2021). 34580318 10.1038/s41598-021-96031-1 PMC8476526

[52] Lee, L. H. et al. An enhanced support vector machine classification framework by using euclidean distance function for text document categorization. *Appl. Intell.* **37**, 80–99 (2012).

[53] Mastrogiuseppe, C. & Moreno-Bote, R. Deep imagination is a close to optimal policy for planning in large decision trees under limited resources. *Sci. Rep.* **12**(1), 10411 (2022). 35729320 10.1038/s41598-022-13862-2 PMC9213460

[54] Li, Z. et al. Measuring and classifying IP usage scenarios: A continuous neural trees approach. *Sci. Rep.* **14**(1), 5144 (2024). 38429421 10.1038/s41598-024-55750-x PMC10907583

[55] Ashraf, W. M. et al. Strategic-level performance enhancement of a 660 MWe supercritical power plant and emissions reduction by AI approach. *Energy Convers. Manage.* **250**, 114913 (2021).

[56] Di Nunno, F., de Marinis, G. & Granata, F. Short-term forecasts of streamflow in the UK based on a novel hybrid artificial intelligence algorithm. *Sci. Rep.* **13**(1), 7036 (2023). 37120698 10.1038/s41598-023-34316-3 PMC10148819

[57] Arjmandi, M. et al. Evaluating algorithms of decision tree, support vector machine and regression for anode side catalyst data in proton exchange membrane water electrolysis. *Sci. Rep.* **13**(1), 20309 (2023). 37985795 10.1038/s41598-023-47174-w PMC10662483

[58] Nakhaei-Kohani, R. et al. Modeling solubility of CO2–N2 gas mixtures in aqueous electrolyte systems using artificial intelligence techniques and equations of state. *Sci. Rep.* **12**(1), 3625 (2022). 35256623 10.1038/s41598-022-07393-z PMC8901744

[59] AlDahoul, N. et al. Suspended sediment load prediction using long short-term memory neural network. *Sci. Rep.* **11**(1), 7826 (2021). 33837236 10.1038/s41598-021-87415-4 PMC8035216

[60] Villeta, M. et al. Efficient optimisation of machining processes based on technical specifications for surface roughness: application to magnesium pieces in the aerospace industry. *Int. J. Adv. Manuf. Technol.* **60**, 1237–1246 (2012).

[61] Shokrani, A., Dhokia, V. & Newman, S. T. Investigation of the effects of cryogenic machining on surface integrity in CNC end milling of Ti–6Al–4V titanium alloy. *J. Manuf. Process.* **21**, 172–179 (2016).

[62] Lalwani, D., Mehta, N. & Jain, P. Experimental investigations of cutting parameters influence on cutting forces and surface roughness in finish hard turning of MDN250 steel. *J. Mater. Process. Technol.* **206**(1–3), 167–179 (2008).

[63] Akhtar, M. N. et al. Optimization of process parameters in CNC turning of aluminum 7075 alloy using L27 array-based Taguchi method. *Materials* **14**(16), 4470 (2021). 34442992 10.3390/ma14164470 PMC8401492

[64] Murat, D. et al. Surface roughness analysis of greater cutting depths during hard turning. *Mater. Test.* **59**(9), 795–802 (2017).

[65] Zheng, G. et al. Effect of cutting parameters on wear behavior of coated tool and surface roughness in high-speed turning of 300M. *Measurement* **125**, 99–108 (2018).

[66] Akgün, M. & Kara, F. Analysis and optimization of cutting tool coating effects on surface roughness and cutting forces on turning of AA 6061 alloy. *Adv. Mater. Sci. Eng.* **2021**(1), 6498261 (2021).

[67] Bhushan, R. K. Multi-response optimization of parameters during turning of AA7075/SiC composite for minimum surface roughness and maximum tool life. *Silicon* **13**, 2845–2856 (2021).

[68] Bhushan, R. K. Minimising tool wear by optimisation (ANOVA) of cutting parameters in machining of 7075Al Alloy SiC particle composite. *Aust. J. Mech. Eng.* **21**(2), 499–517 (2023).

[69] Selvakumar, S., Sreebalaji, V. & Ravikumar, K. Machinability analysis and optimization in micro turning on tool wear for titanium alloy. *Mater. Manuf. Processes* **36**(7), 792–802 (2021).

